# Design, Synthesis, DFT Study and Antifungal Activity of Pyrazolecarboxamide Derivatives

**DOI:** 10.3390/molecules21010068

**Published:** 2016-01-08

**Authors:** Jin-Xia Mu, Yan-Xia Shi, Ming-Yan Yang, Zhao-Hui Sun, Xing-Hai Liu, Bao-Ju Li, Na-Bo Sun

**Affiliations:** 1 Department of Environmental Engineering, China Jiliang University, Hangzhou 310018, China; 2 College of Chemical Engineering, Zhejiang University of Technology, Hangzhou 310014, China; shiyanxia@caas.cn; 3 Institute of Vegetables and Flowers, Chinese Academy of Agricultural Sciences, Beijing 100014, China; yangmingyanzjut@163.com (M.-Y.Y.); sunzhaohuizjut@163.com (Z.-H.S.); 4 College of Biology and Environmental Engineering, Zhejiang Shuren University, Hangzhou 310015, China; nabosun@gmail.com

**Keywords:** pyrazole, DFT, synthesis, antifungal activity, SAR

## Abstract

A series of novel pyrazole amide derivatives were designed and synthesized by multi-step reactions from phenylhydrazine and ethyl 3-oxobutanoate as starting materials, and their structures were characterized by NMR, MS and elemental analysis. The antifungal activity of the title compounds was determined. The results indicated that some of title compounds exhibited moderate antifungal activity. Furthermore, DFT calculations were used to study the structure-activity relationships (SAR).

## 1. Introduction

**Figure 1:**
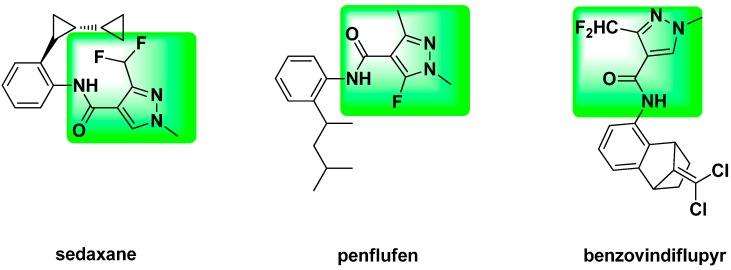
The commercial pyrazole amide fungicides.

Heterocyclic structures are important key features in natural products or synthetic medicines and pesticides because of their high-efficiency, low toxicity and diversity of possible substituents [1,2,3,4,5]. This has become a hot research topic in the medicine and pesticides field. Pyrazole is an important kind of heterocyclic nitrogen compound [6,7,8], which derivatives exhibit a wide range of biological activities, such as antifungal [9], insecticidal [10], herbicidal [11], anticancer [12], anti-inflammatory [13] and so on. So far, many pyrazole derivatives such as the insecticides tebufenpyrad and chlorantraniliprole, the fungicides penthiopyrad and pyraclostrobin, and the medicine antipyrine, *etc.*, have been successfully developed by different companies*.* On the other hand, heterocycles with amide groups are reported as a class of compounds displaying extensive biological activities, such as anti-biofilm [14], herbicidal [15,16], anticancer [17], antifungal [18], antiproliferative [19], plant growth regulation [20] and so on. They represent an important class of natural and synthetic products and extremely versatile building blocks for the manufacture of bioactive compounds in pharmaceutical drug design and the agrochemical industry. In recent years, many succinate-dehydrogenase-inhibitor (SDHI) fungicides had been introduced into the market for effective treatment of fruit and vegetable crops, such as sedaxane, penflufen and benzovindiflupyr (Figure 1). Penflufen is one of the members in this new class of fungicides for the treatment of a wide range of diseases. From Figure 1, the structure of penflufen contains a phenyl ring, an amide group and a 1,3-dimethyl-5-fluoropyrazole moiety.

**Scheme 1:**
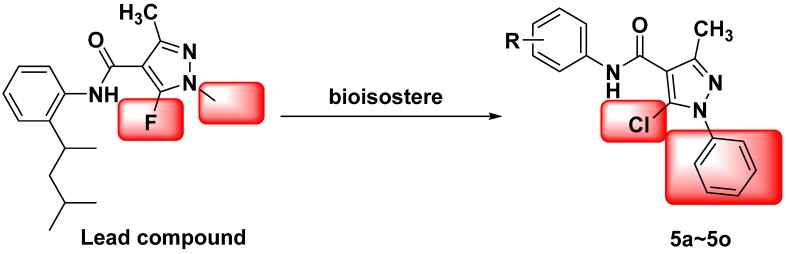
Design strategy of the title compounds.

In our previous work, some amide derivatives exhibited excellent herbicidal [21] and fungicidal activity [22]. In line with our continued efforts to synthesize bioactive lead compounds for crop protection [23,24,25,26,27,28,29], the title amide compounds had modified N-substituted pyrazole pharmacophore scaffolds. It is reported that the halogens exhibit similar biological effects. Meanwhile, the aromatic ring also held diverse functions. In order to discover highly active pyrazole amide compounds, the commercial amide fungicide penflufen was selected as a lead compound, and the 1-methyl and 5-fluoro groups on the pyrazole ring were replaced by a phenyl ring and chloro group, respectively. Our original strategy is depicted in Scheme 1. It is possible that pyrazole amide derivatives possess antifungal activities.

## 2. Results and Discussion

### 2.1. Synthesis and Spectra

In the present paper, a series of pyrazole amide analogues were designed by replacing methyl group and fluorine of penflufen with phenyl and chlorine. First, the pyrazole ring was synthesized from phenylhydrazine and ethyl acetoacetate as starting materials using a classical Knorr reaction, then, using the universal Vilsmeier-Haack reaction, apyrazole with an aldehyde group was obtained in excellent yield. The COOH group can be prepared easily through oxidation with KMnO_4_. The target compounds were prepared according our previous work [30]. When 5-chloro-3-methyl-1-phenyl-1*H*-pyrazole-4-carbonyl chloride was reacted with a substituted amine, organic base Et_3_N was used instead of the inorganic base K_2_CO_3_, while the temperature must be maintained at 0–5 °C, as higher temperatures decreased the yield of product. The imide or lactam product will be obtained.

All the compounds were identified and characterized by ^1^H-NMR, ^13^C-NMR, MS and elemental analysis. In the ^1^H-NMR spectra of target compounds, all the -NH proton signals can be found around 7.59–10.88 ppm. The appearance of a signal around at 2.5 ppm is assigned to the methyl of the pyrazole ring. Meanwhile, most of the title compounds exhibited the M−H^−^ peak in the ESI-MS results.

### 2.2. Antifungal Activities

The *in vivo* fungicidal results of the title compounds against *Pythium ultimum* Trow, *Phytophthora infestans* (Mont.) De Bary, *Corynespora cassiicola, Botrytis cinerea* and *Rhizoctonia solani* are listed in Table 1. Zhongshengmycin, dimethomorph, fludioxonil, chlorothalonil and validamycin were used as controls. From Table 1, some of the pyrazole compounds such as compounds **5a** (77.78%), **5d** (55.56%), **5e** (66.67%), **5h** (66.67%), **5i** (44.44%) and **5l** (77.78%) exhibited good control efficacy against *Pythium ultimum* Trow at a concentration of 100 μg/mL. These compounds show better activity against *Pythium ultimum* Trow than that of the control. Some of them on the other hand showed low activity (below 40%) against *Pythium ultimum* Trow, and some of them can’t inhibit *Pythium ultimum* Trow. For example, compounds **5b** (−11.11%), **5f** (−55.56%) and **5k** (−88.89%) had no inhibitory activity against *Pythium ultimum* Trow. On the contrary, these compounds increased the fungal growth. The control zhongshengmycin also can’t inhibit the fungus *Pythium ultimum* Trow. Among the new compounds, compounds **5d** (75.33%) and **5h** (75.89%) exhibited excellent control efficacy against *Corynespora cassiicola*, which was better than that of control chlorothalonil (45.9%), while compounds **5a** (44.49%), **5c** (48.41%) and **5g** (46.17%) displayed the same control efficacy as chlorothalonil (45.9%). None of the title compounds exhibited any inhibition effect against *Botrytis cinerea* and *Phytophthora infestans* (Mont.) De Bary, except for compound **5h**, which displayed weak inhibition (21.38%) of *Botrytis cinerea*. For the fungus *Rhizoctonia solani*, most of them had no inhibitory activity, although compound **5a** showed a control efficacy of 61.11%, which was similar to that of the most active control fungicide validamycin (62.5%).

**Table 1:** The antifungal activity of the title compounds *in vivo* at 100 ppm (%).

No.	*Pythium ultimum*	*Phytophthora infestans*	*Corynespora cassiicola*	*Botrytis cinerea*	*Rhizoctonia solani*
**5a**	77.78	−0.80	44.49	−9.97	61.11
**5b**	−11.11	5.92	21.21	−17.69	35.00
**5c**	11.11	5.36	48.41	−10.94	31.67
**5d**	55.56	0.04	75.33	−52.42	0.00
**5e**	66.67	−0.80	23.46	−29.27	0.00
**5f**	−55.56	−0.80	34.39	−11.90	0.00
**5g**	22.22	1.44	46.17	−45.67	0.00
**5h**	66.67	−0.80	75.89	21.38	0.00
**5i**	44.44	6.76	6.92	−13.35	0.00
**5j**	22.22	−0.80	32.71	−32.16	0.00
**5k**	−88.89	−0.80	−0.93	−55.31	0.00
**5l**	77.78	−0.80	−0.93	−36.98	0.00
Zhongshengmycin	0.0				
Dimethomorph		97.8			
Chlorothalonil			45.9		
Fludioxonil				86.98	
Validamycin					62.5

Note: *Pythium ultimum* Trow for tomato, *Phytophthora infestans*(Mont.) De Bary for tomato, *Corynespora cassiicola* for cucumber, *Botrytis cinerea* for cucumber and *Rhizoctonia solani* for cucumber; All the data were determined three times.

### 2.3. DFT Calculation and SAR

The total molecular energy and frontier orbital energy levels of compound **5h** and penflufen are listed in Table 2. Energy gap between HOMO and LUMO was calculated by B3LYP.

**Figure 2:**
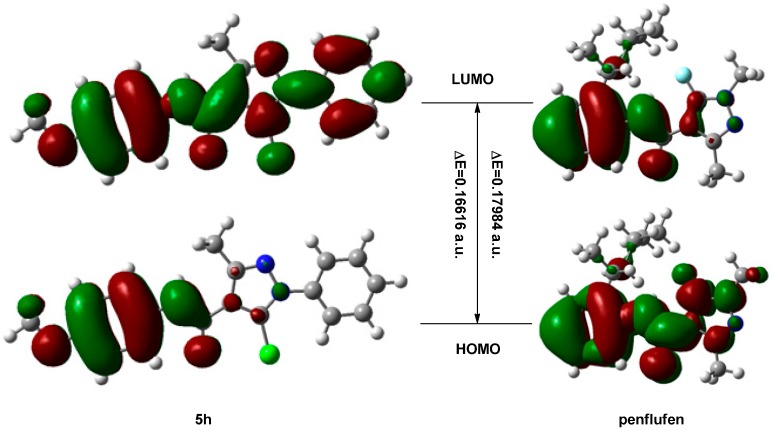
Frontier molecular orbitals of compound **5h** and penflufen.

According to the frontier molecular orbital theory, the HOMO and LUMO are the most important factors that affect the bioactivity [31,32]. HOMO has the priority to provide electrons, while the LUMO can accept electrons first. Thus a study of the frontier orbital energy can provide useful information about the biological mechanism of action. We selected the compound **5h** with the best antifungal activity among the title compounds and the commercial drug penflufen as models to compare their frontier molecular orbital. Taking the DFT results for example, the geometry of the framework of the compound **5h** is hardly influenced by the introduction of either the phenyl ring or the pyrazole ring (Figure 2). The HOMO of the title compound is mainly located on the 3-Me phenyl ring, pyrazole ring and amide bond, while, the LUMO of the title compound is located on the pyrazole ring, 3-Me phenyl ring, phenyl ring, chlorine atom and amide bond. On the other hand, the HOMO of the penflufen is mainly located on the phenyl ring, pyrazole ring, fluorine atom, methyl group and amide bond, while the LUMO of the penflufen is located on the pyrazole ring, phenyl ring and amide bond. From Figure 2, the electron transition ocurrs from the 3-methylbenzene ring, amide bond and pyrazole ring to the *N*-phenyl ring in compound **5h**, while the energy gap between the HOMO and LUMO is 0.16616 Hartree. On the contrary, the electron transition from the phenyl ring, pyrazole ring, fluorine atom, methyl group and amide bond to the phenyl ring, pyrazole ring and amide bond in the compound Penflufen, while the energy gap between the HOMO and LUMO is 0.17984 Hartree. The differences between the two compounds are the electron transition orientation and energy gap. The fact that the title compound has strong affinity suggests the importance of the frontier molecular orbital in the π-π stacking or hydrophobic interactions. This also implies that substituted of phenyl ring had an important impact on the antifungal activity. Furthermore, the MO combination provided meaningful clues as to the structural features of this new family of fungicides that will be helpful in the design of more potent compounds in the future: first the methyl group in the 3-position of pyrazole ring had no impact on the antifungal activity; second for the halo group in the 5-position, a higher group negativity is better; and third, the amide bond is necessary.

**Table 2:** Total energy and frontier orbital energy.

DFT	5 h	Penflufen
*E*_total_/Hartree ^b^	−1394.96053044	−1039.42133553
*E*_HOMO_/Hartree	−0.21495	−0.21213
*E*_LUMO_/Hartree	−0.04879	−0.03229
Δ*E* ^a^/Hartree	0.16616	0.17984

^a^ Δ*E* = *E*_LUMO_−*E*_HOMO_; ^b^ 1 Hartree = 4.35974417 × 10^−18^ *J* = 27.2113845 ev.

**Figure 3:**
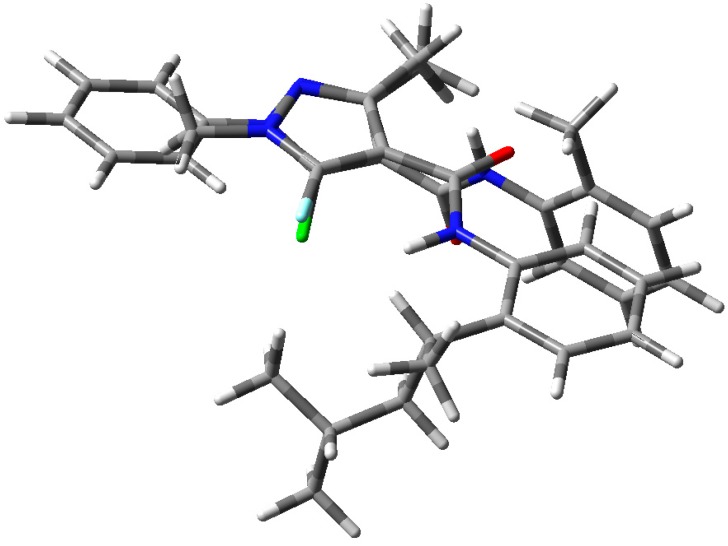
Overlay of energy-minimized structures of **5h** and penflufen.

The structural difference is also an important issue. For example, the amide group orientation between the two compounds is opposite (Figure 3). We therefore speculate that this is the confirmation required between the phenyl ring and pyrazole ring when they bind to the target receptor.

## 3. Experimental Section

### 3.1. General Information

All the chemical reagents were analytical grade or prepared in our lab. Melting points were measured using an X-4 apparatus (Taike, Beijing, China) and were uncorrected. ^1^H-NMR and ^13^C-NMR spectra were recorded on an Avance 500 MHz spectrometer (Bruker, Fallanden, Switzerland) using CDCl_3_ as solvent. Mass spectra were determined on a LCQ Advantage LC/mass detector instrument (Thermo Finnigan, Silicon Valley, CA, USA). Elemental analysis data of the title compounds were obtainned by a 240C analyzer (Perkin-Elmer, Waltham, MA, USA).

### 3.2. Synthesis

*3-Methyl-1-phenyl-1H-pyrazol-5(4H)-one* (**1**). Ethyl acetoacetate (13.0 g, 100 mmol) was added to a solution of phenylhydrazine (10.8 g, 100 mmol) in ethanol (20 mL), then the mixture was refluxed for 4 h, then the ethanol was removed under reduced pressure to give compound **1** as a yellow solid (14.3 g, yield 82.6%,m.p.: 125–126 °C).

*5-Chloro-3-methyl-1-phenyl-1H-pyrazole-4-carbaldehyde* (**2**) [33]. Phosphorus oxychloride (250 mmol) was added dropwise into *N*,*N*-dimethylformamide (100 mmol) at 0–5 °C. After the mixture was stirred for 30 min, 3-methyl-1-phenyl-1*H*-pyrazol-5(4*H*) one (**1**, 5.22 g, 30 mmol) was added portionwise. Then then mixture was heated to 120 °C for another 1 h. The reaction mixture was poured slowly into crushed ice, and the precipitated solid was filtered and dried, to give **2** as a light yellow solid (5.61 g, yield: 85.0%, m.p.: 136–137 °C).

*5-Chloro-3-methyl-1-phenyl-1H-pyrazole-4-carboxylic acid* (**3**) [34]. 5-Chloro-3-methyl-1-phenyl-1*H*-pyrazole-4-carbaldehyde (**2**, 5.5 g, 25 mmol) and potassium permanganate (4.74g, 30 mmol) were added to water (50 mL) and refluxed under microwave irradiation for 0.5 h. The reaction mixture was filtered, acidified to pH = 1 using HCl, to give 3 as a white solid that was filtered off and dried (5.6 g, yield: 95%,m.p.: 230–231 °C).

*5-Chloro-3-methyl-1-phenyl-1H-pyrazole-4-carbonyl chloride* (**4**) [35]. To 5-chloro-3-methyl-1-phenyl-1*H*-pyrazole-4-carboxylic acid (**3**, 7.50 mmol) thionyl chloride (30 mmol) was added and the mixture was refluxed for 2 h. After the reaction is completed, the excess of thionyl chloride were evaporated to give **4** as a yellow liquid that was used without further purification.

#### General Procedure for the Preparation of Pyrazole Amide Compounds **5a**–**m**

To a solution of 5-chloro-3-methyl-1-phenyl-1*H*-pyrazole-4-carbonyl chloride (**4**, 7 mmol) and Et_3_N (7.5 mmol) in THF (10 mL), a substituted aniline (7.50 mmol) was added dropwise under 0–5 °C for 1 h. Then the mixture was vigorously stirred at ambient temperature for 8 h, then evaporated under reduced pressure, and subsequently the mixture was exacted with EtOAc. The organic layer was dried over MgSO_4_ and evaporated. The residue was purified by chromatography on a silica gel column using petroleum ether (60–90 °C) and ethyl acetate as the eluents to afford the title compounds. All the compounds were synthesized according to this procedure (Scheme 2). All the data can be found in Supplementary Materials (see Figures S1–S33 for more details).

**Scheme 2:**
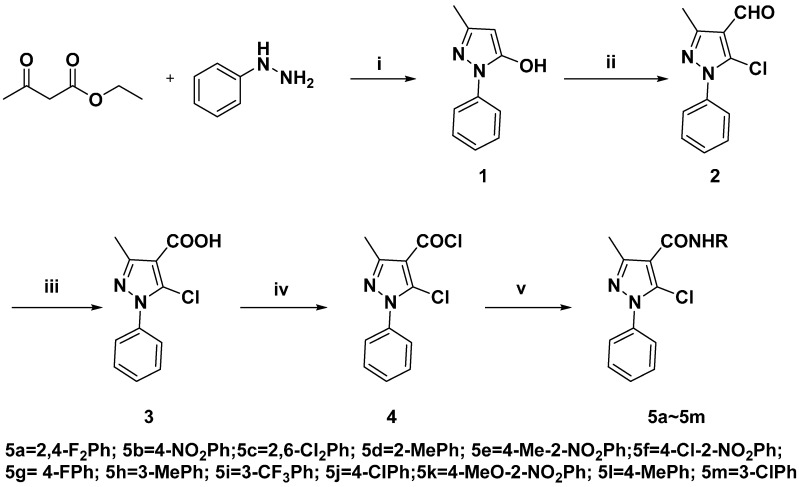
The synthetic route of title compounds. *Reagents and Condition*: i. EtOH, reflux, 4 h, 83%; ii. POCl_3_/DMF, 0–5 °C to 120 °C, 1.5 h, 85%; iii. a. KMnO_4_ H_2_O, MW, 0.5 h; b. HCl, 95%; iv. SOCl_2_, reflux, 2 h; v. RNH_2_, THF, Et_3_N, r.t., 8 h.

*5-Chloro-N-(2,4-difluorophenyl)-3-methyl-1-phenyl-1H-pyrazole-4-carboxamide* (**5a**). Yellow solid, m.p. 138–139 °C, yield 94%, ^1^H-NMR δ: 2.62 (s, 3H, CH_3_), 6.89–6.94 (m, 2H, Ph-H), 7.49–7.51 (m, 1H, Ph-H), 7.53–7.54 (m, 4H, Ph-H), 8.21 (s, 1H, NH), 8.38–8.44 (m, 1H, Ph-H); ^13^C-NMR δ: 14.66, 103.57, 111.19, 111.36, 112.79, 122.86, 122.93, 125.54, 126.12, 129.20, 129.27, 137.31, 152.34, 159.78; ESI-MS: 346.04 [M − H]^−^, 348.01 [M − H + 2]^+^, 347.97 [M + H]^+^, 349.97 [M + H + 2]^+^; Elemental anal. calculated for C_17_H_12_ClF_2_N_3_O (%): C, 58.72; H, 3.48; N, 12.08; found: C, 58.90; H, 3.17; N, 12.00.

*5-Chloro-3-methyl-N-(4-nitrophenyl)-1-phenyl-1H-pyrazole-4-carboxamide* (**5b**). Yellow solid, m.p. 159–161 °C, yield 98%, ^1^H-NMR δ: 2.62 (s, 3H, CH_3_), 7.51–7.55 (m, 5H, Ph-H), 7.83–7.85 (m, 2H, Ph-H), 8.25–8.27 (d, *J* = 8.0 Hz, 2H, Ph-H), 8.38 (s, 1H, NH); ESI-MS: 355.04 [M − H]^−^, 356.97 [M − H + 2]^+^, 356.97 [M + H]^+^, 358.98 [M + H + 2]^+^; Elemental anal. calculated for C_17_H_13_ClN_4_O_3_ (%): C, 57.23; H, 3.67; N, 15.70; found: C, 57.44; H, 3.57; N, 15.65.

*5-Chloro-N-(2,6-dichlorophenyl)-3-methyl-1-phenyl-1H-pyrazole-4-carboxamide* (**5c**). Yellow solid, m.p. 151–153 °C, yield 99%, ^1^H-NMR δ: 2.61 (s, 3H, CH_3_), 7.20–7.24 (m, 1H, Ph-H), 7.42–7.43 (d, *J* = 4.0 Hz, 2H, Ph-H), 7.48–7.55 (m, 5H, Ph-H), 7.76 (s, 1H, NH); ESI-MS: 379.91 [M + H]^+^, 381.92 [M + H + 2]^+^; Elemental anal. For C_17_H_12_Cl_3_N_3_O (%), calculated: C, 53.64; H, 3.18; N, 11.04; found: C, 53.56; H, 3.17; N, 11.32.

*5-Chloro-3-methyl-1-phenyl-N-(o-tolyl)-1H-pyrazole-4-carboxamide* (**5d**). Yellow solid, m.p. 132–133 °C, yield 38.4%, ^1^H-NMR δ: 2.36 (s, 3H, CH_3_), 2.62 (s, 3H, CH_3_), 7.09–7.13 (m, 1H, Ph-H), 7.22–7.24 (d, *J* = 8.0 Hz, 2H, Ph-H), 7.49–7.54 (m, 5H, Ph-H), 7.85 (s, 1H, NH), 8.05–8.07 (m, 1H, Ph-H); ^13^C-NMR δ: 14.65, 18.13, 113.34, 122.87, 125.08, 125.53, 126.81, 128.61, 129.06, 129.20, 130.50, 135.66, 137.40, 152.40, 159.90; ESI-MS: 324.04 [M − H]^−^, 326.11 [M − H + 2]^+^, 326.01 [M + H]^+^, 327.99 [M + H + 2]^+^; Elemental anal. calculated for C_18_H_16_ClN_3_O (%): C, 66.36; H, 4.95; N, 12.90; found: C, 66.43; H, 4.89; N, 13.02.

*5-Chloro-3-methyl-N-(4-methyl-2-nitrophenyl)-1-phenyl-1H-pyrazole-4-carboxamide* (**5e**). Yellow solid, m.p. 187–188 °C, yield 71.5%, ^1^H-NMR δ: 2.42 (s, 3H, CH_3_), 2.61 (s, 3H, CH_3_), 7.48–7.50 (m, 1H, Ph-H), 7.51–7.53 (m, 5H, Ph-H), 8.04 (s, 1H, Ph-H), 8.71–8.73 (m, 1H, Ph-H), 10.81 (s, 1H, NH); ESI-MS: 368.98 [M − H]^−^ , 370.95 [M − H + 2]^+^, 371.01 [M + H]^+^ , 373.00 [M + H + 2]^+^; Elemental anal. For C_18_H_15_ClN_4_O_3_ (%), calculated: C, 58.31; H, 4.08; N, 15.11; found: C, 58.23; H, 4.17; N, 14.99.

*5-Chloro-N-(4-chloro-2-nitrophenyl)-3-methyl-1-phenyl-1H-pyrazole-4-carboxamide* (**5f**). Yellow solid, m.p. 163–165 °C, yield 96%, ^1^H-NMR δ: 2.63 (s, 3H, CH_3_), 6.79–6.81 (m, 1H, Ph-H), 7.51–7.53 (m, 5H, Ph-H), 8.25–8.26 (m, 1H, Ph-H), 8.88–8.91 (m, 1H, Ph-H), 10.88 (s, 1H, NH); ESI-MS: 389.01 [M − H]^−^, 390.97 [M − H + 2]^+^, 392.91 [M − H + 4]^+^, 391.05 [M + H]^+^, 393.01 [M − H + 2]^+^ , 394.99 [M + H + 4]^+^; Elemental anal. calculated for C_17_H_12_Cl_2_N_4_O_3_ (%): C, 52.19; H, 3.09; N, 14.32; found: C, 52.33; H, 3.13; N, 14.15.

*5-Chloro-N-(4-fluorophenyl)-3-methyl-1-phenyl-1H-pyrazole-4-carboxamide* (**5g**). Yellow solid, m.p. 154–156 °C, yield 62.2%, ^1^H-NMR δ: 2.61 (s, 3H, CH_3_), 7.06–7.10 (m, 2H, Ph-H), 7.50–7.55 (m, 5H, Ph-H), 7.59–7.61 (m, 2H, Ph-H), 7.59 (s, 1H, NH); ESI-MS: 328.06 [M − H]^−^, 330.10 [M − H + 2]^+^, 329.96 [M + H]^+^, 332.02 [M + H + 2]^+^; Elemental anal. calculated for C_17_H_13_ClFN_3_O (%): C, 61.92; H, 3.97; N, 12.74; found: C, 61.90; H, 4.17; N, 12.65.

*5-Chloro-3-methyl-1-phenyl-N-(m-tolyl)-1H-pyrazole-4-carboxamide* (**5h**). Yellow solid, m.p. 78–81 °C, yield 99%, ^1^H-NMR δ: 2.37 (s, 3H, CH_3_), 2.61 (s, 3H, CH_3_), 6.96–6.98 (m, 1H, Ph-H), 7.23–7.26 (m, 2H, Ph-H), 7.36–7.38 (d, *J* = 8.0Hz, 1H, Ph-H), 7.51–7.53 (m, 5H, Ph-H), 7.91 (s, 1H, NH); ^1^^3^C-NMR δ: 14.55, 21.49, 113.36, 117.26, 120.84, 125.41, 125.54, 128.90, 129.08, 129.24, 137.41, 137.53, 139.07, 152.18, 159.91; ESI-MS: 324.08 [M − H]^−^, 326.01 [M − H + 2]^+^, 326.00 [M + H]^+^, 328.03 [M + H + 2]^+^; Elemental anal. calculated for C_18_H_16_ClN_3_O (%): C, 66.36; H, 4.95; N, 12.90; found: C, 66.58; H, 4.86; N, 12.13. 

*5-Chloro-3-methyl-1-phenyl-N-(3-(trifluoromethyl)phenyl)-1H-pyrazole-4-carboxamide* (**5i**). Yellow solid, m.p. 97–98 °C, yield 72.5%, ^1^H-NMR δ: 2.61 (s, 3H, CH_3_), 7.39–7.41 (m, 1H, Ph-H), 7.47–7.49 (m, 1H, Ph-H), 7.50–7.54 (m, 5H, Ph-H), 7.80–7.82 (m, 1H, Ph-H), 7.95 (s, 1H, Ph-H), 8.14 (s, 1H, NH); ESI-MS: 378.02 [M − H]^−^, 380.00 [M − H + 2]^+^, 380.02 [M + H]^+^, 381.93 [M+H+2]^+^; Elemental anal. calculated for C_18_H_13_ClF_3_N_3_O (%): C, 58.72; H, 3.48; N, 12.08; found: C, 58.59; H, 3.45; N, 12.12.

*5-Chloro-N-(4-chlorophenyl)-3-methyl-1-phenyl-1H-pyrazole-4-carboxamide* (**5j**). Yellow solid, m.p. 161–162 °C, yield 78.1%, ^1^H-NMR δ: 2.52 (s, 3H, CH_3_), 7.24–7.26 (m, 2H, Ph-H), 7.43–7.45 (m, 5H, Ph-H), 7.49–7.51 (m, 2H, Ph-H), 7.89 (s, 1H, NH); ESI-MS: 343.92 [M − H]^−^, 345.89 [M − H + 2]^+^, 345.91 [M + H]^+^, 347.92 [M + H + 2]^+^; Elemental anal. calculated for C_17_H_13_Cl_2_N_3_O (%): C, 58.98; H, 3.78; N, 12.14; found: C, 59.11; H, 3.95; N, 12.19. 

*5-Chloro-N-(4-methoxy-2-nitrophenyl)-3-methyl-1-phenyl-1H-pyrazole-4-carboxamide* (**5k**). Yellow solid, m.p. 157–159 °C, yield 66%, ^1^H-NMR δ: 2.61 (s, 3H, CH_3_), 3.88 (s, 3H, CH_3_), 7.28–7.29 (m, 1H, Ph-H), 7.53–7.55 (m, 5H, Ph-H), 7.70 (s, 1H, Ph-H), 8.71–8.74 (m, 1H, Ph-H), 10.69 (s, 1H, NH) ; ESI-MS: 385.07 [M − H]^−^, 387.14 [M − H + 2]^+^, 386.95 [M + H]^+^, 389.03 [M+H+2]^+^; Elemental anal. calculated for C_18_H_15_ClN_4_O_4_ (%): C, 55.89; H, 3.91; N, 14.49; found: C, 55.98; H, 4.17; N, 14.38.

*5-Chloro-3-methyl-1-phenyl-N-(p-tolyl)-1H-pyrazole-4-carboxamide* (**5l**). Yellow solid, m.p. 115–116 °C, yield 73.7%, ^1^H-NMR δ: 2.34 (s, 3H, CH_3_), 2.60 (s, 3H, CH_3_), 7.16–7.18 (m, 2H, Ph-H), 7.47–7.49 (m, 2H, Ph-H), 7.51–7.53 (m, 5H, Ph-H), 7.90 (s, 1H, NH); ESI-MS: 324.03 [M − H]^−^, 326.02 [M − H + 2]^+^, 326.02 [M + H]^+^, 328.05 [M + H + 2]^+^; Elemental anal. calculated for C_18_H_16_ClN_3_O (%): C, 66.36; H, 4.95; N, 12.90; found: C, 66.48; H, 5.13; N, 12.88.

*5-Chloro-N-(3-chlorophenyl)-3-methyl-1-phenyl-1H-pyrazole-4-carboxamide* **5m**. Yellow solid, m.p. 81–82 °C, yield 88%, ^1^H-NMR δ: 2.60 (s, 3H, CH_3_), 7.11–7.14 (m, 1H, Ph-H), 7.28–7.30 (m, 1H, Ph-H), 7.44–7.46 (m, 1H, Ph-H), 7.52–7.53 (m, 5H, Ph-H), 7.77 (s, 1H, Ph-H), 7.99 (s, 1H, NH); ESI-MS: 343.98 [M − H]^−^, 345.98 [M − H + 2]^+^, 345.96 [M + H]^+^, 347.93 [M + H + 2]^+^; Elemental anal. calculated for C_17_H_13_Cl_2_N_3_O (%): C, 58.98; H, 3.78; N, 12.14; found: C, 59.05; H, 3.87; N, 12.33.

### 3.3. Antifungal Activity

The antifungal activity of compounds **5a**–**5m** against *Pythium ultimum* Trow, *Phytophthora infestans* (Mont.) De Bary, *Corynespora cassiicola, Botrytis cinerea* and *Rhizoctonia solani* was evaluated according to reference [36,37]. A potted plant test method was adopted. Germination was induced by soaking cucumber seeds in water for 2 h at 50 °C and then keeping the seeds moist for 24 h at 28 °C in an incubator. When the radicles were 0.5 cm, the seeds were grown in plastic pots containing a 1:1 (*v*/*v*) mixture of vermiculite and peat. Cucumber plants used for inoculations were at the stage of two cotyledons, and tomato plants were five euphyllas. Tested compounds and commercial fungicides were sprayed with a hand sprayer on the surface of the leaves and on a fine morning, at the standard concentration of 100 μg/mL, each plant was sprayed with compounds and commercial fungicides (200 μL). Dimethomorph, fludioxonil, chlorothalonil, validamycin, and zhongshengmycin were used as controls. After 2 h, inoculations of *Phytophthora infestans*, *Corynespora cassiicola* and *Botrytis cinerea* were carried out by spraying fungal spore suspension with 1 × 10^4^ spore/mL, inoculation of *Rhizoctonia solani* and *Pythium ultimum* were carried out by spraying mycelial suspension of 2 × 10^4^ CFU/mL, which was smashed with a T10 basic ULTRA-TURRAX^®^ (IKA, Guangzhou, China). Each kind of inoculum was sprayed at 300 μL/plant. Each treatment was replicated four times. After inoculation, the plants were maintained at 18–30 °C (mean temperature of 24 °C and above 80% relative humidity (RH)). The antifungal activity was evaluated when the non-treated plant (blank) fully developed symptoms. The area of inoculated treated leaves covered by disease symptoms was assessed and compared to that of nontreated ones to determine the average disease index. The relative control efficacy of compounds compared to the blank assay was calculated via the following equation:

relative control efficacy (%) = (*CK−PT*)/*CK* × 100% (1)

where CK is the average disease index during the blank assay and PT is the average disease index after treatment during testing. All experiments were replicated three times.

### 3.4. Theoretical Calculations

According to the above crystal structure, a molecular unit was selected as the initial structure, while the DFT-B3LYP/6-31G (d,p) methods in the Gaussian 03 package [38] were used to optimize the structure of the title compound. Vibration analysis showed that the optimized structures were in accordance with the minimum points on the potential energy surfaces, which means no virtual frequencies, proving that the obtained optimized structures were stable. All the convergent precisions were the system default values, and all the calculations were carried out on the DELL computer.

## 4. Conclusions

Some interesting pyrazole amide derivatives were designed and synthesized. Their structures were confirmed by NMR, MS and elemental analysis. The antifungal evaluation of the newly synthesized pyrazole amide derivatives showed that among the tested compounds 5-chloro-*N*-(2,4-difluorophenyl)-3-methyl-1-phenyl-1*H*-pyrazole-4-carboxamide (**5a**), 5-chloro-3-methyl-1-phenyl-*N*-(o-tolyl)-1*H*-pyrazole-4-carboxamide (**5d**), 5-chloro-3-methyl-*N*-(4-methyl-2-nitrophenyl)-1-phenyl-1*H*-pyrazole-4-carboxamide (**5e**), 5-chloro-3-methyl-1-phenyl-*N*-(m-tolyl)-1*H*-pyrazole-4-carboxamide (**5h**) and 5-chloro-3-methyl-1-phenyl-*N*-(p-tolyl)-1*H*-pyrazole-4-carboxamide (**5l**) showed good antifungal activity against *Pythium ultimum*. Interestingly, compound **5d** and **5h** still exhibited good antifungal activity against *Corynespora cassiicola*. The best activity compound **5h** was selected as a model and its frontier orbitals studied in comparison with the commercial fungicide penflufen.

## Supplementary Materials

Supplementary materials can be accessed at: http://www.mdpi.com/1420-3049/21/1/68/s1.
