# Supplementary material for: Design, Synthesis, DFT Study and Antifungal Activity of Pyrazolecarboxamide Derivatives
Source: Molecules. 2016 Jan 8;21(1):68. doi: 10.3390/molecules21010068 (PMC6274113; doi:10.3390/molecules21010068)
Supplement: Supplementary file 1 [file molecules-21-00068-s001.pdf]

Supplementary Materials: Design, Synthesis, DFT Study and Antifungal Activity of Pyrazolecarboxamide Derivatives

Jin-Xia Mu, Yan-Xia Shi, Ming-Yan Yang, Zhao-Hui Sun, Xing-Hai Liu, Bao-Ju Li and Na-Bo Sun

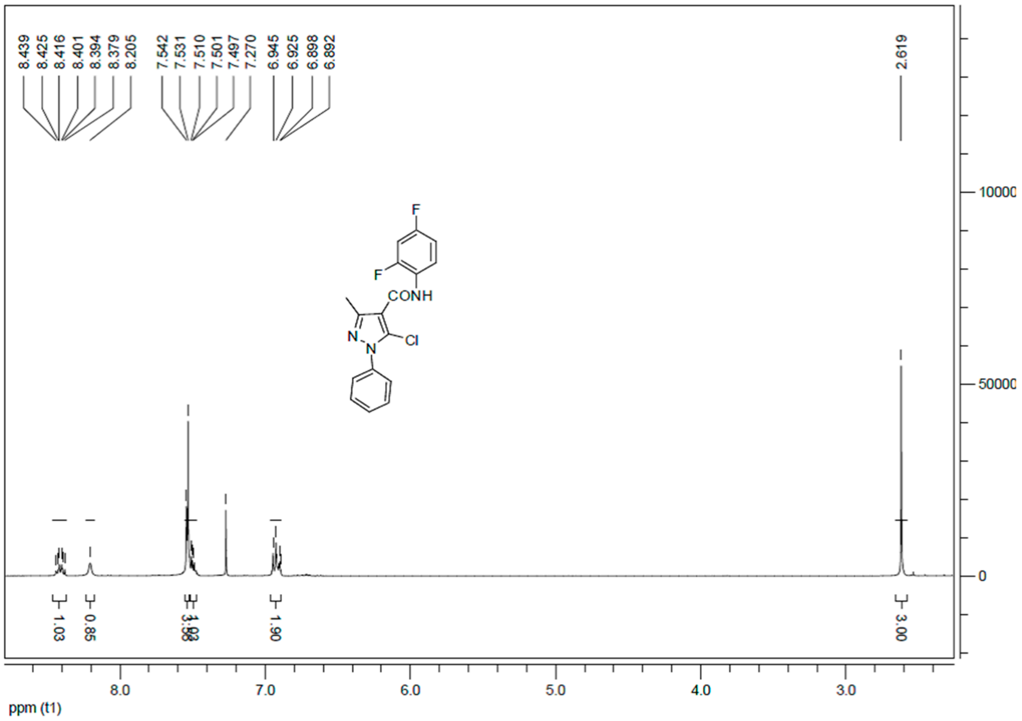

Figure S1. ^{1}H-NMR spectrum of compound **5a**.

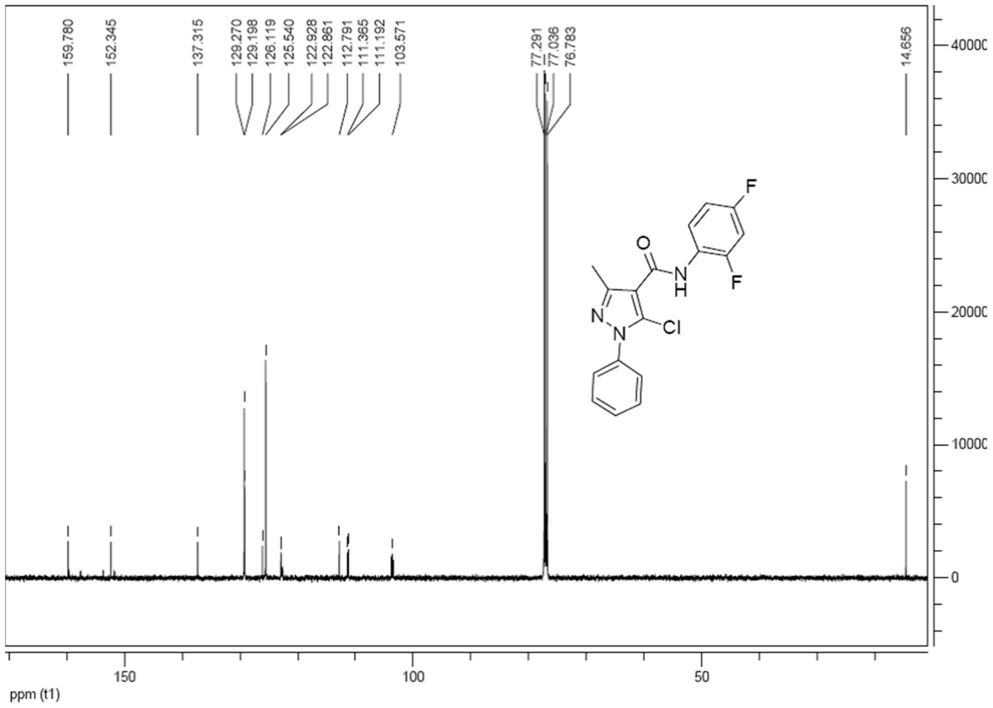

Figure S2. ^{13}C-NMR spectrum of compound **5a**.

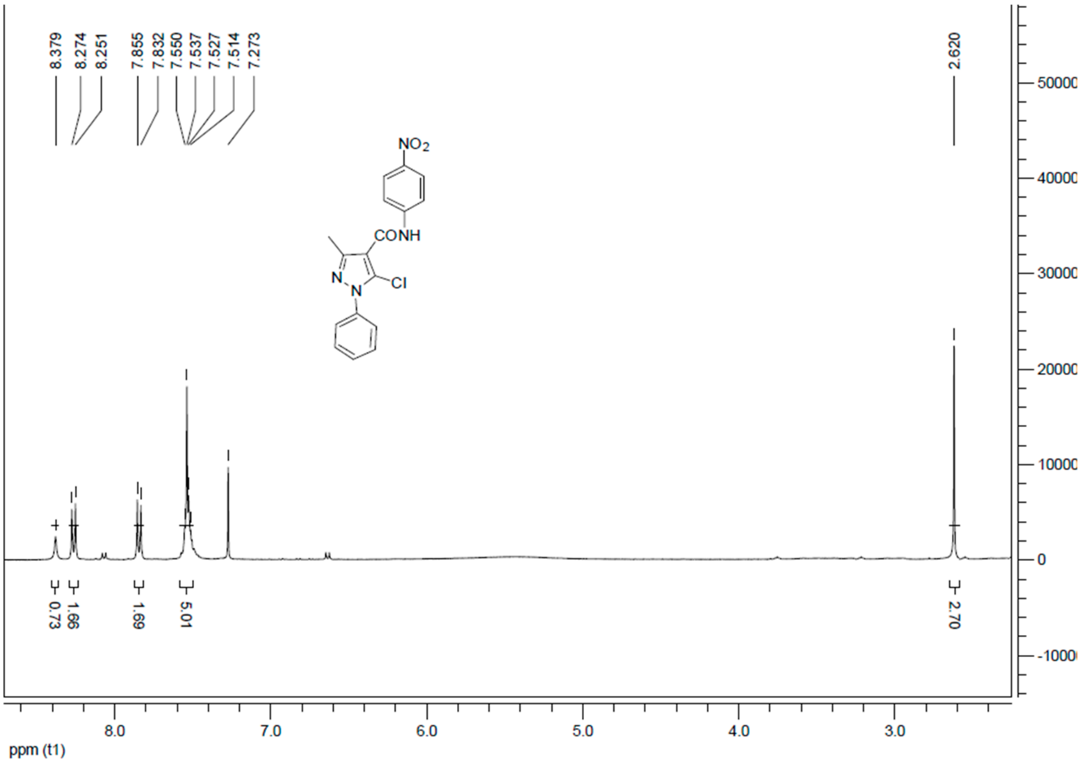

Figure S3. ^{1}H-NMR spectrum of compound **5b**.

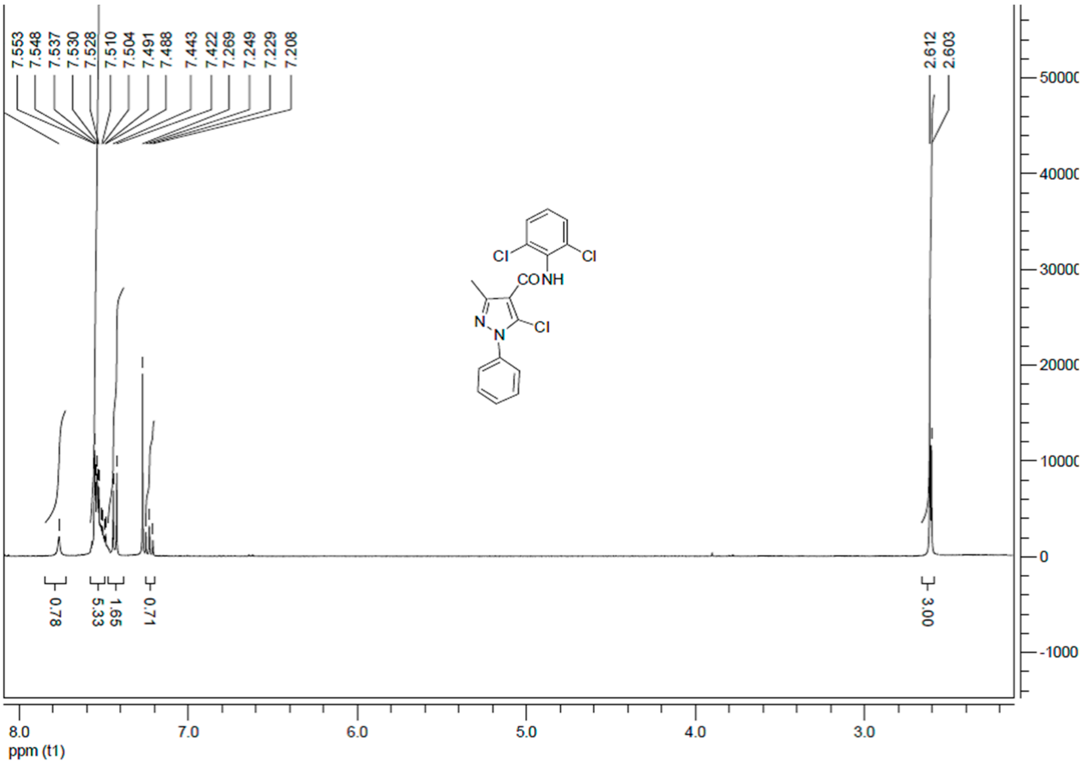

Figure S4. ^{1}H-NMR spectrum of compound **5c**.

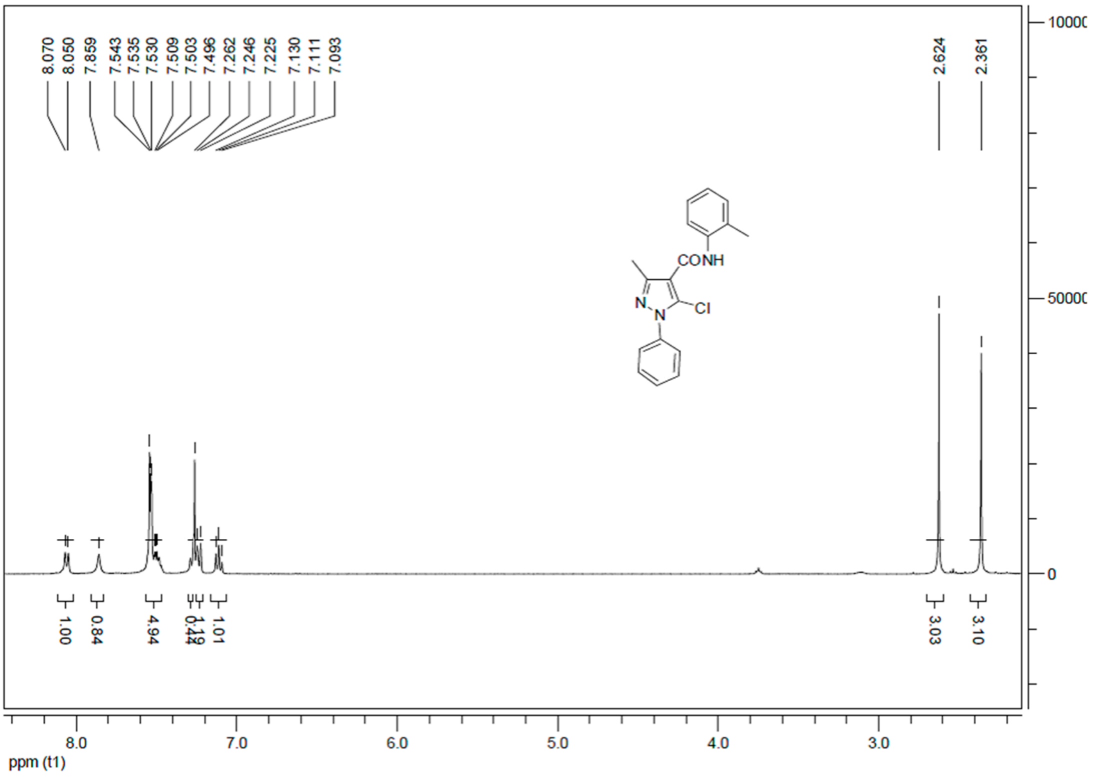

Figure S5. ^{1}H-NMR spectrum of compound **5d**.

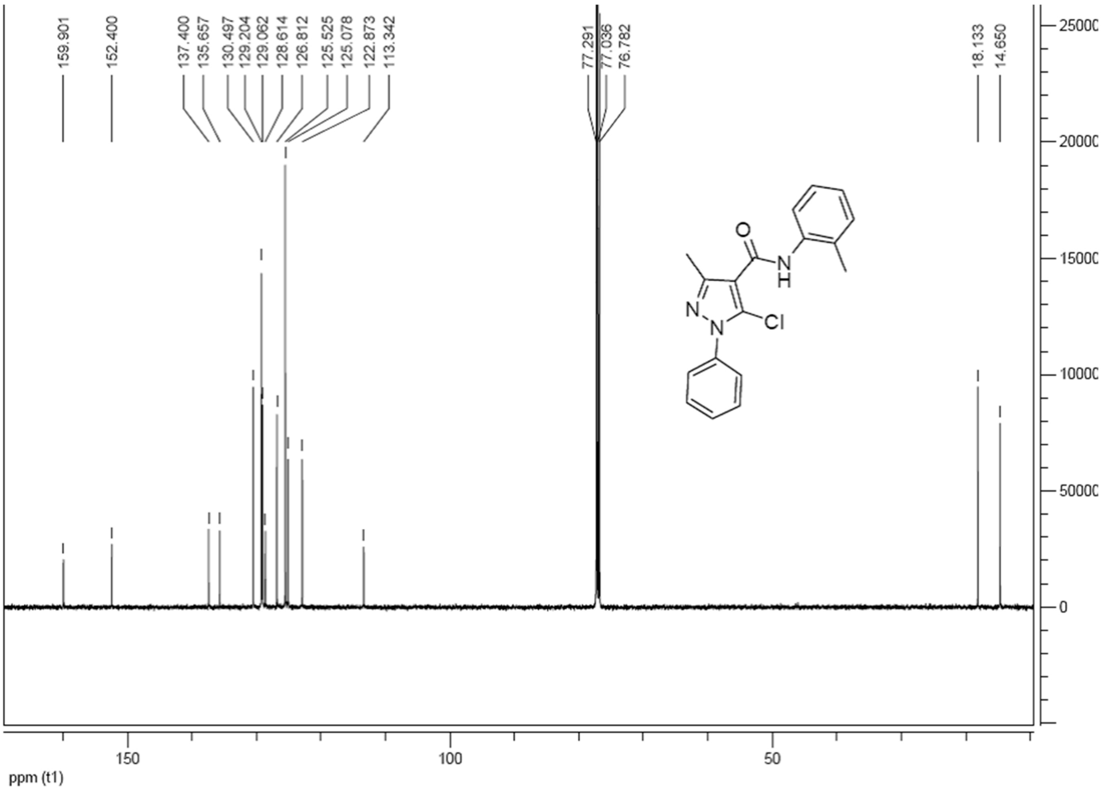

Figure S6. ^{13}C-NMR spectrum of compound **5d**.

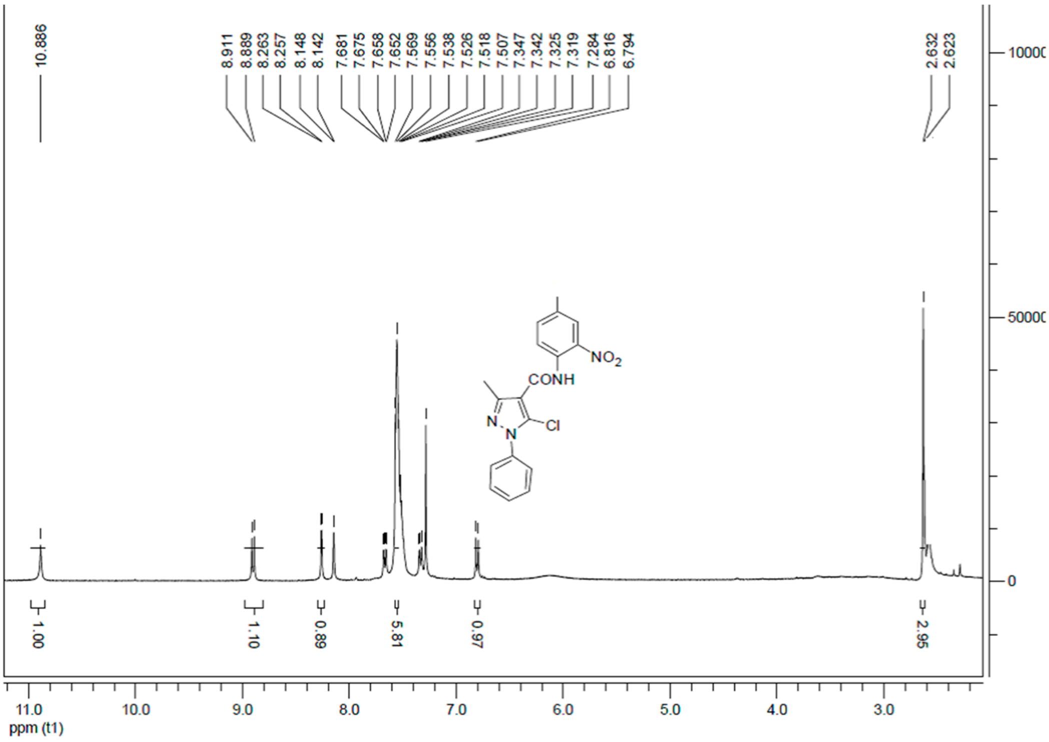

Figure S7. ^{1}H-NMR spectrum of compound **5e**.

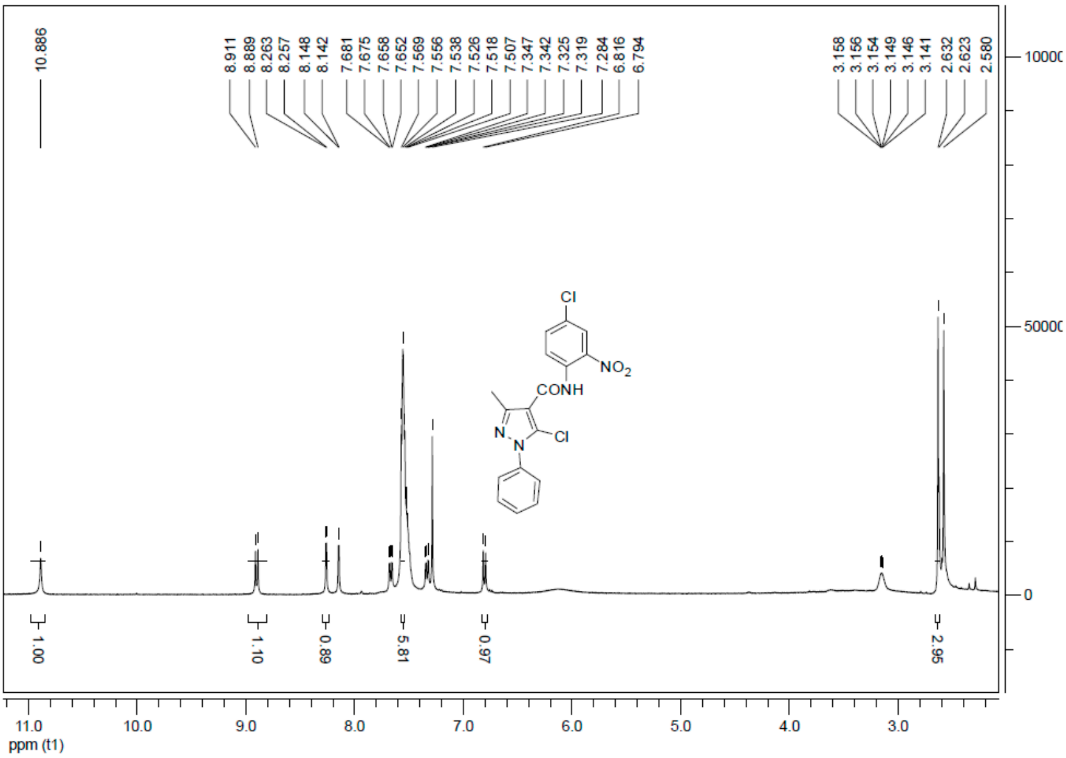

Figure S8. ^{1}H-NMR spectrum of compound **5f**.

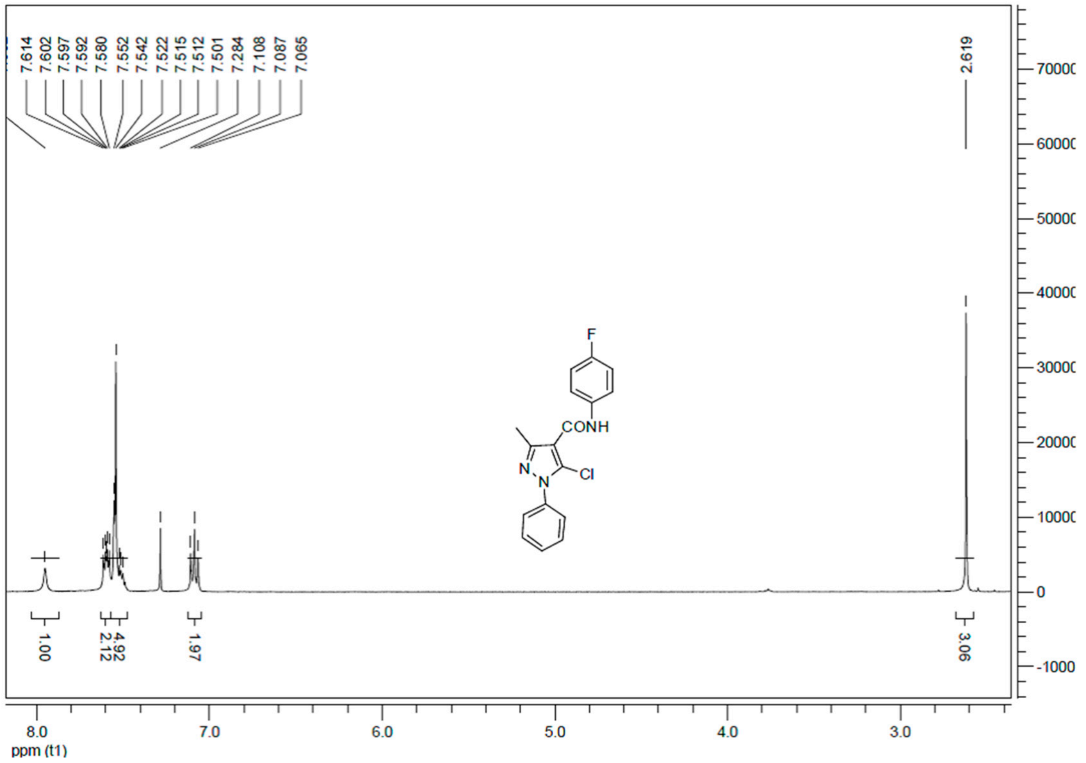

Figure S9. ^{1}H-NMR spectrum of compound **5g**.

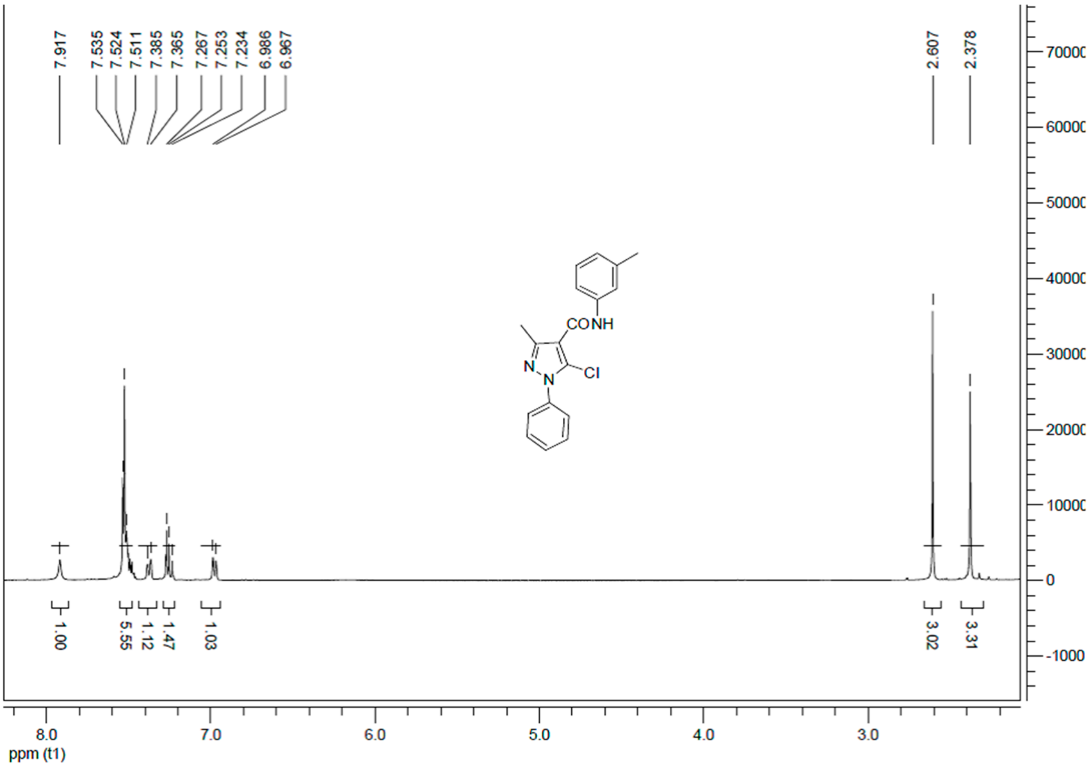

Figure S10. ^{1}H-NMR spectrum of compound **5h**.

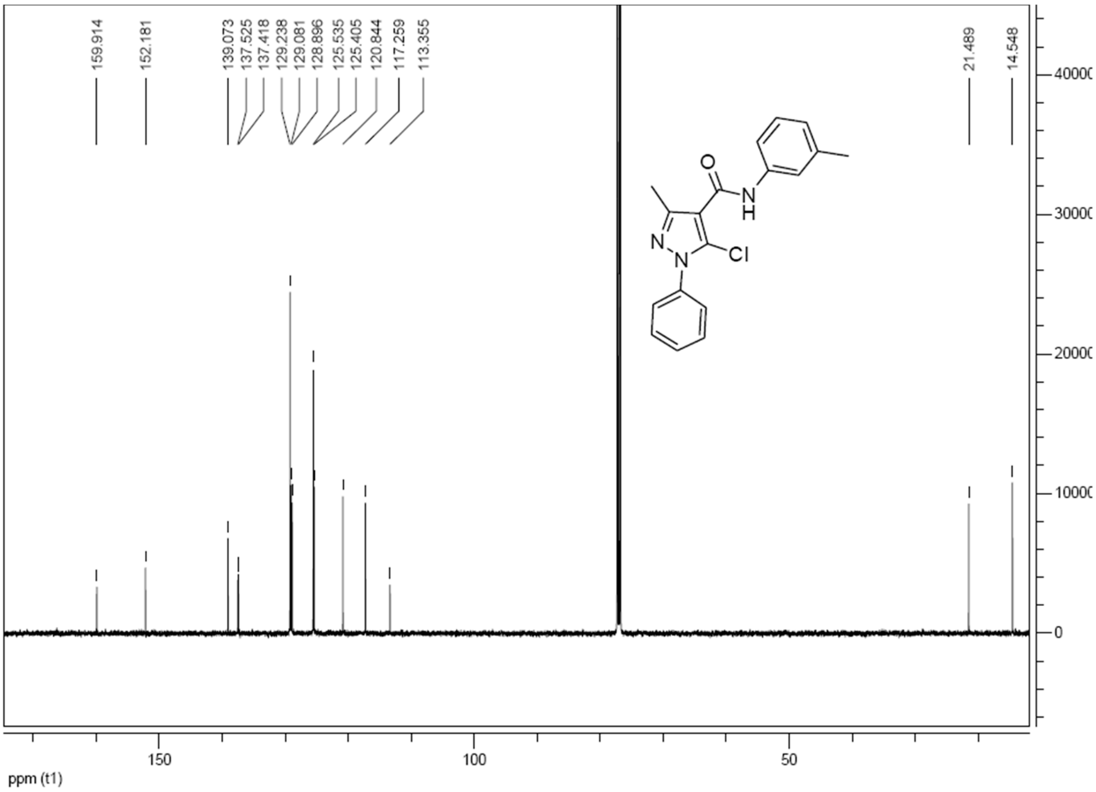

Figure S11. ^{13}C-NMR spectrum of compound **5h**.

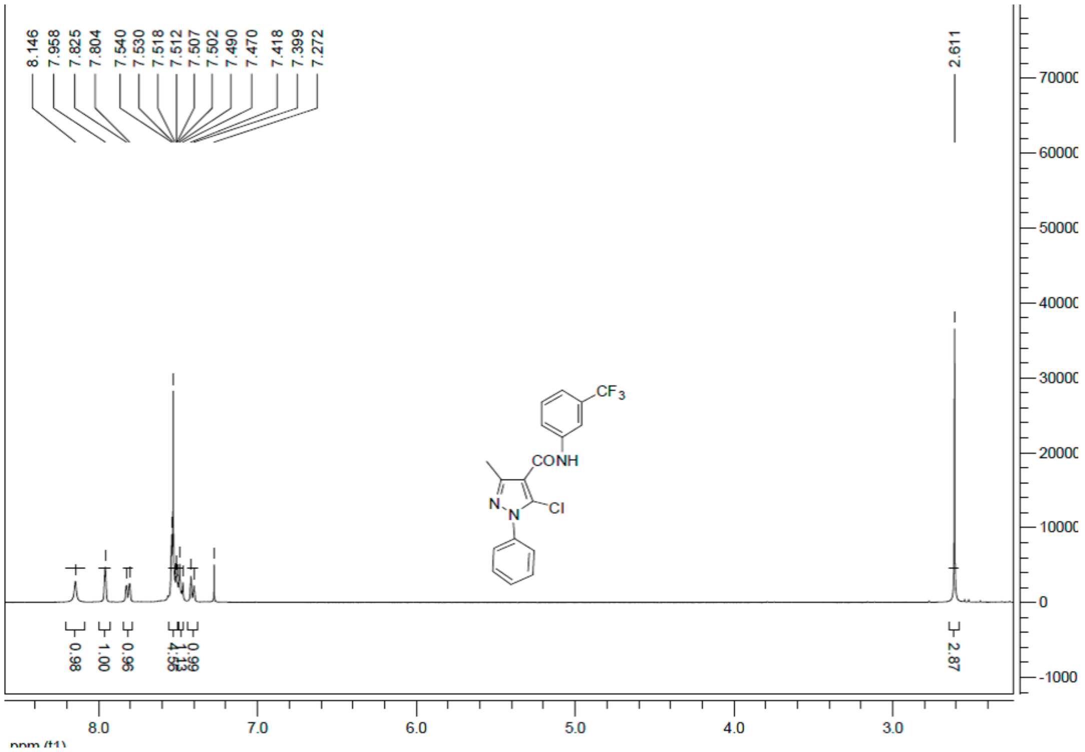

Figure S12. ^{1}H-NMR spectrum of compound **5i**.

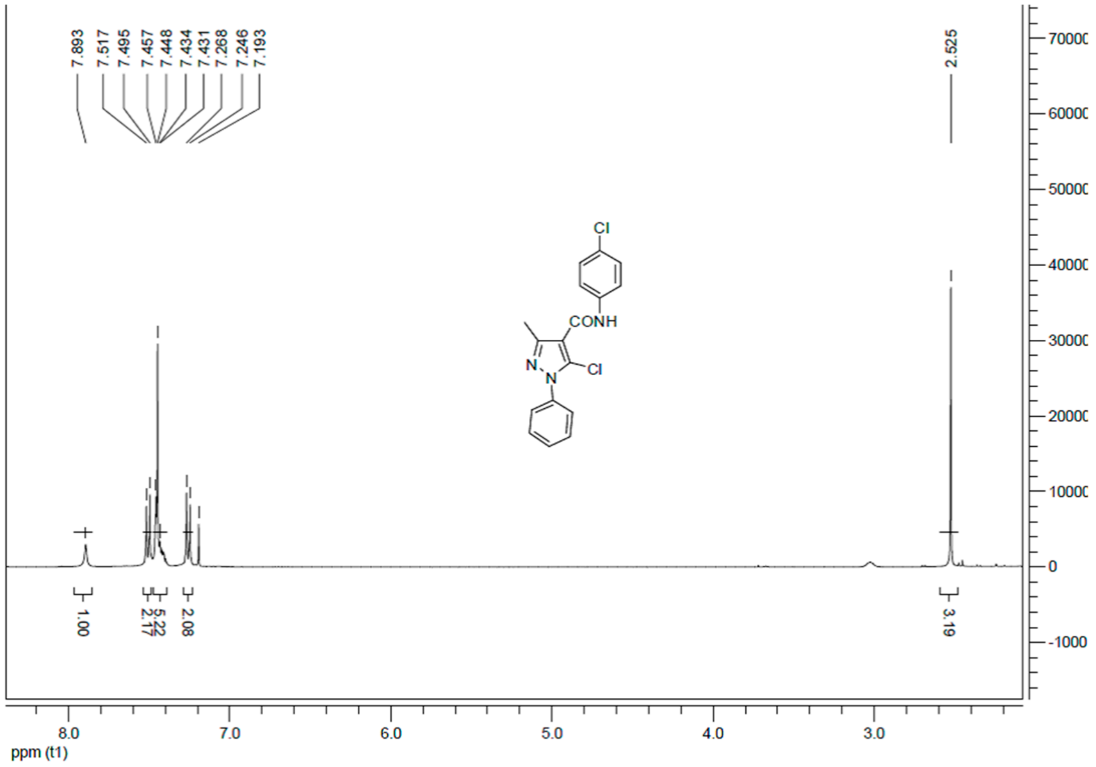

Figure S13. ^{1}H-NMR spectrum of compound **5j**.

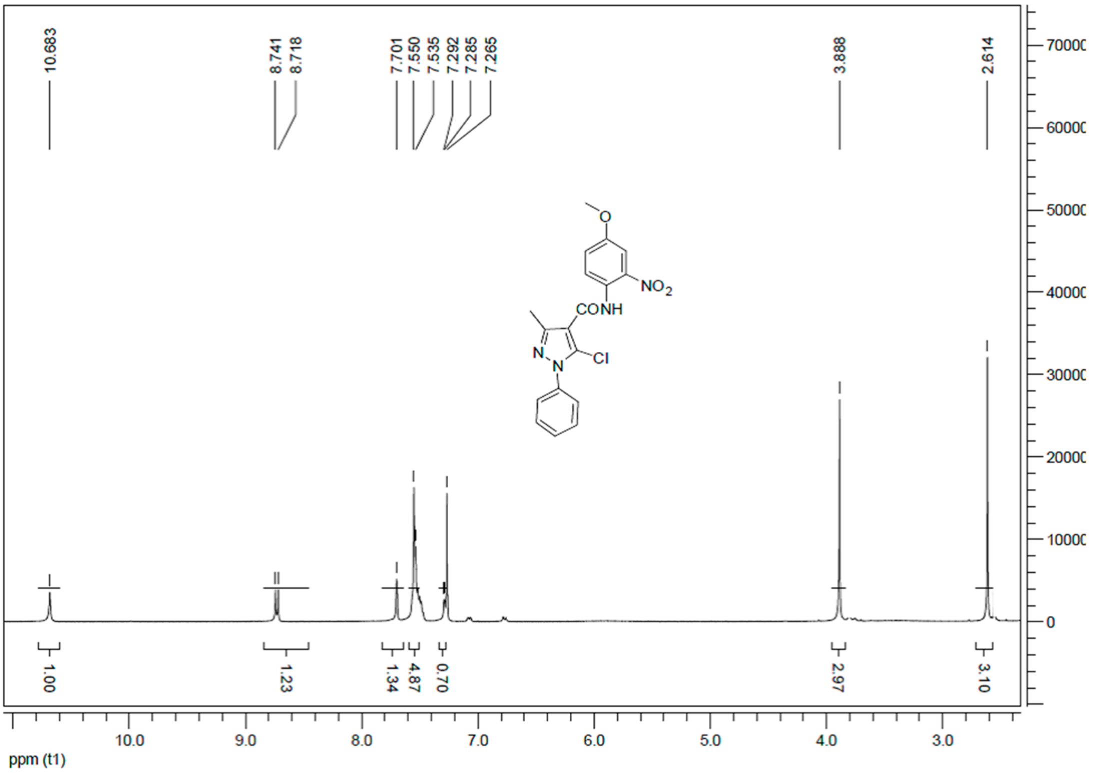

Figure S14. ^{1}H-NMR spectrum of compound **5k**.

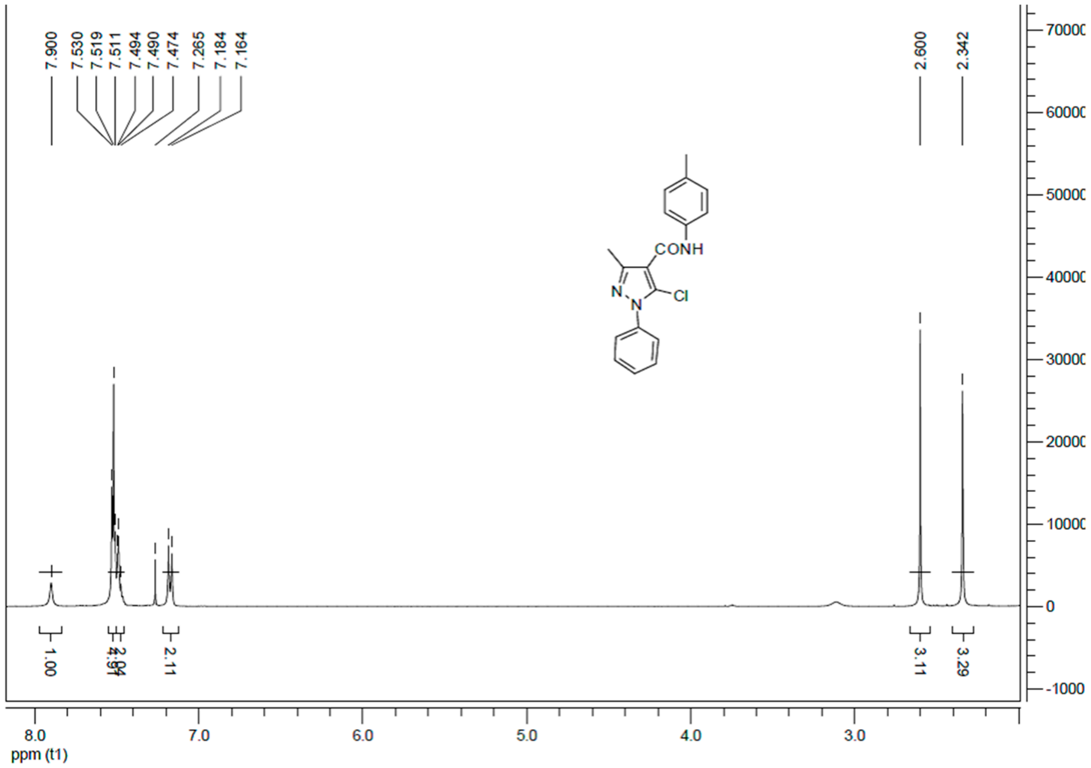

Figure S15. ^{1}H-NMR spectrum of compound **5l.**

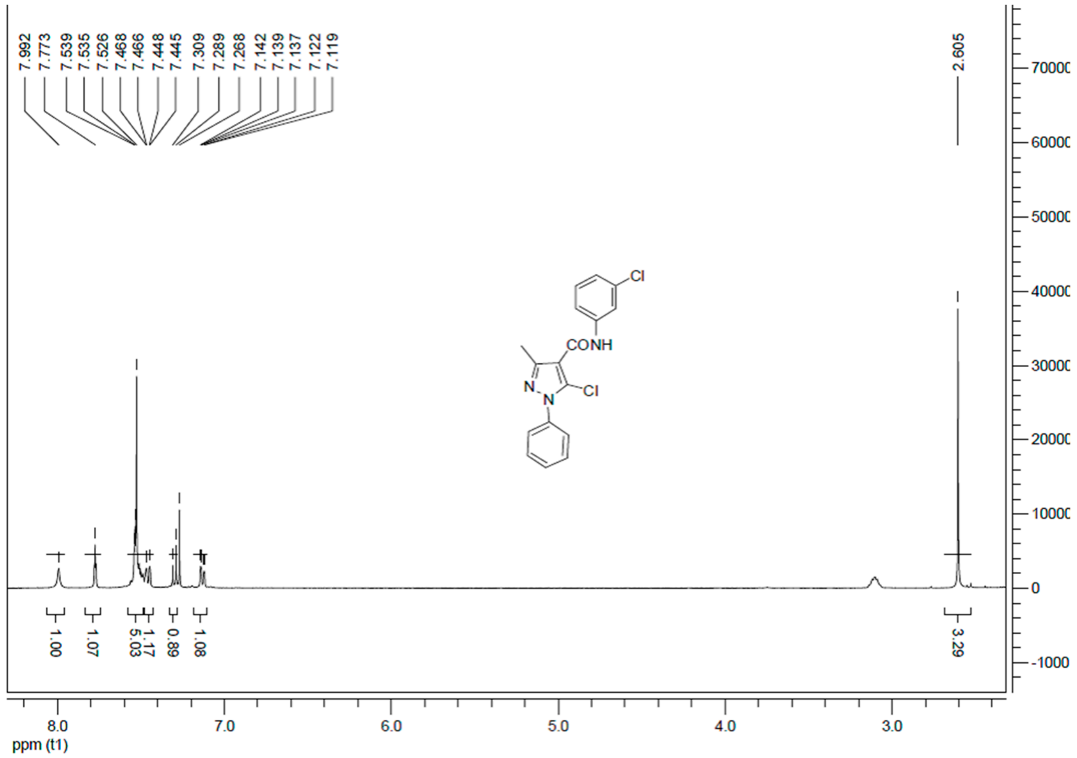

Figure S16. ^{1}H-NMR spectrum of compound **5m.**

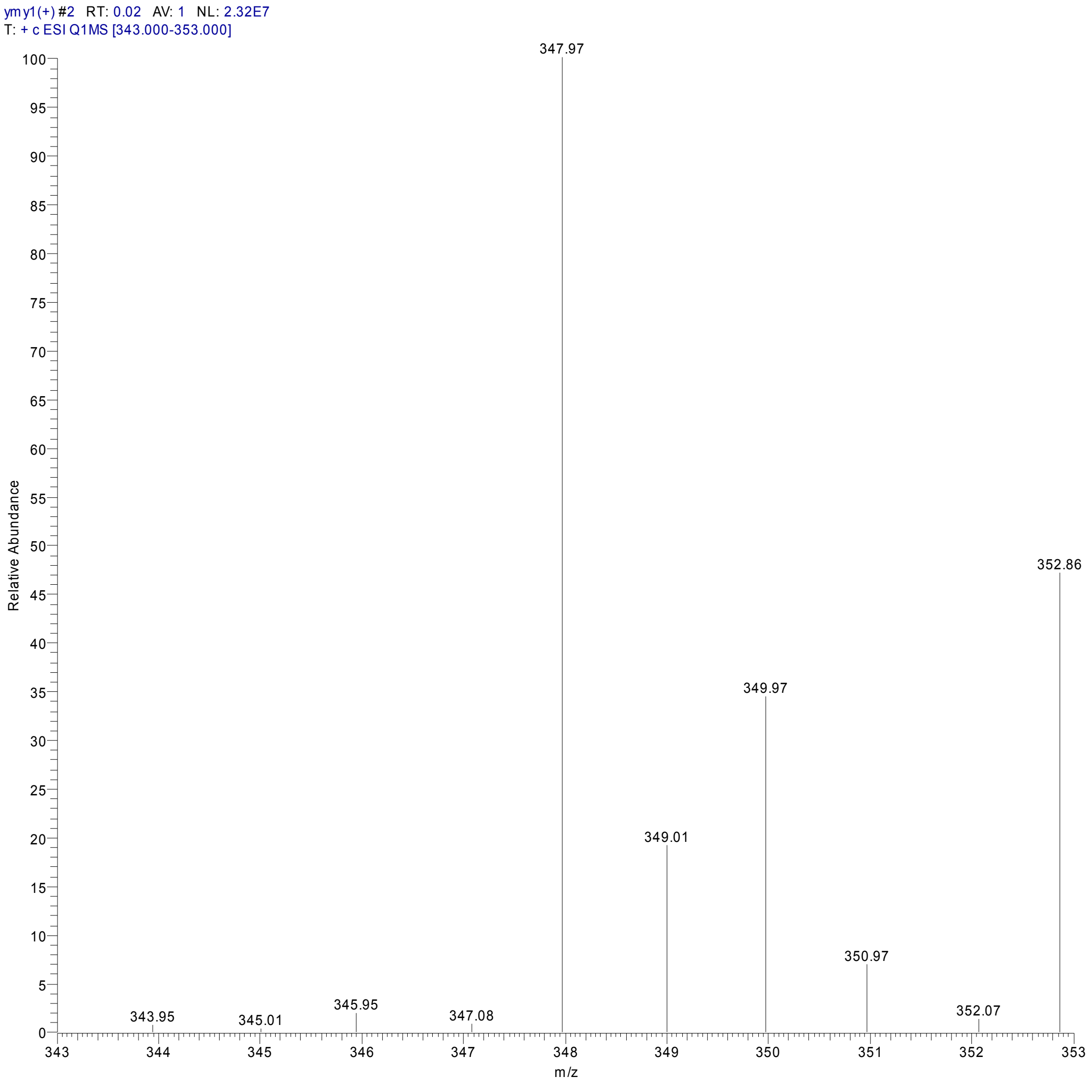

Figure S17. MS spectrum of compound **5a** (+).

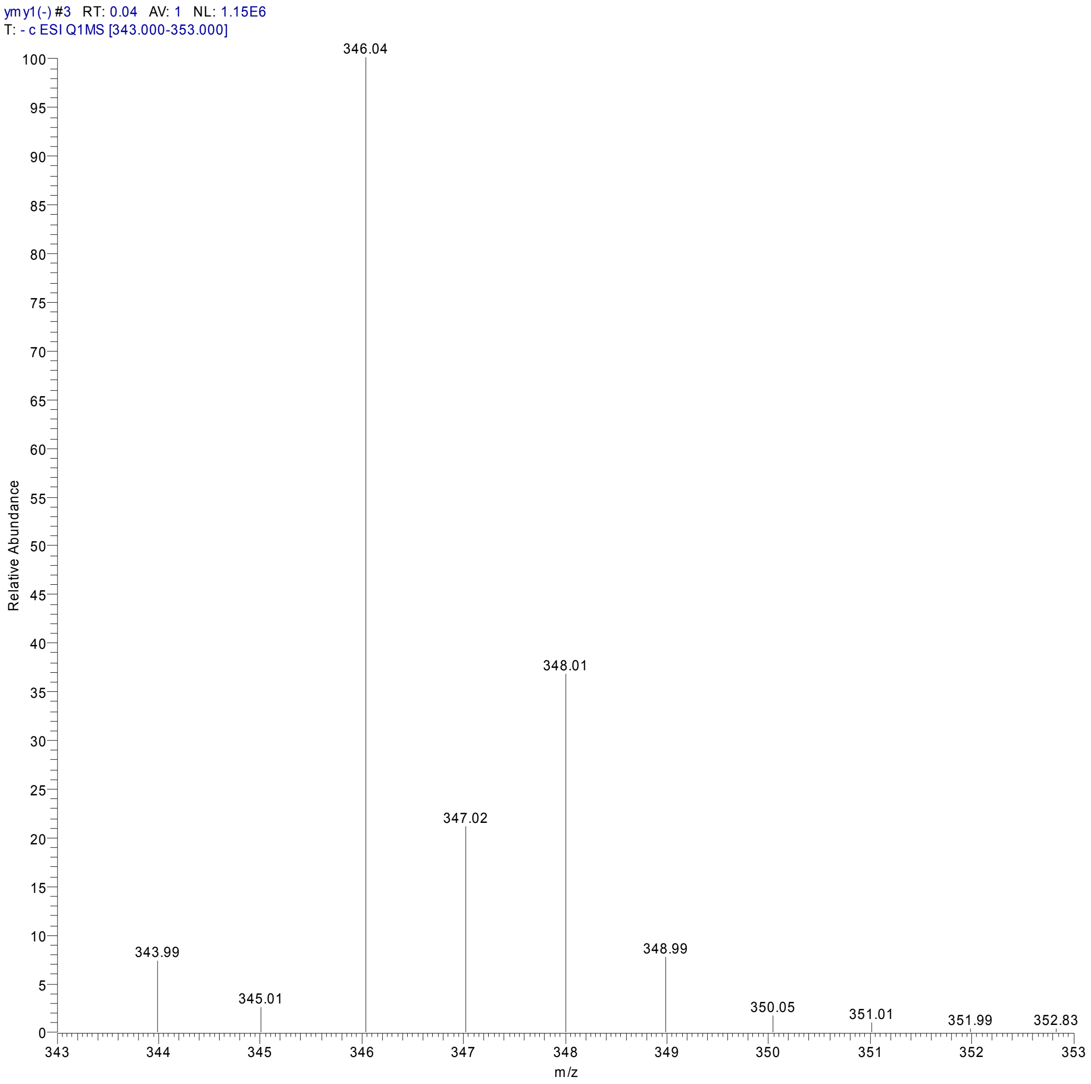

Figure S18. MS spectrum of compound **5a** (−).

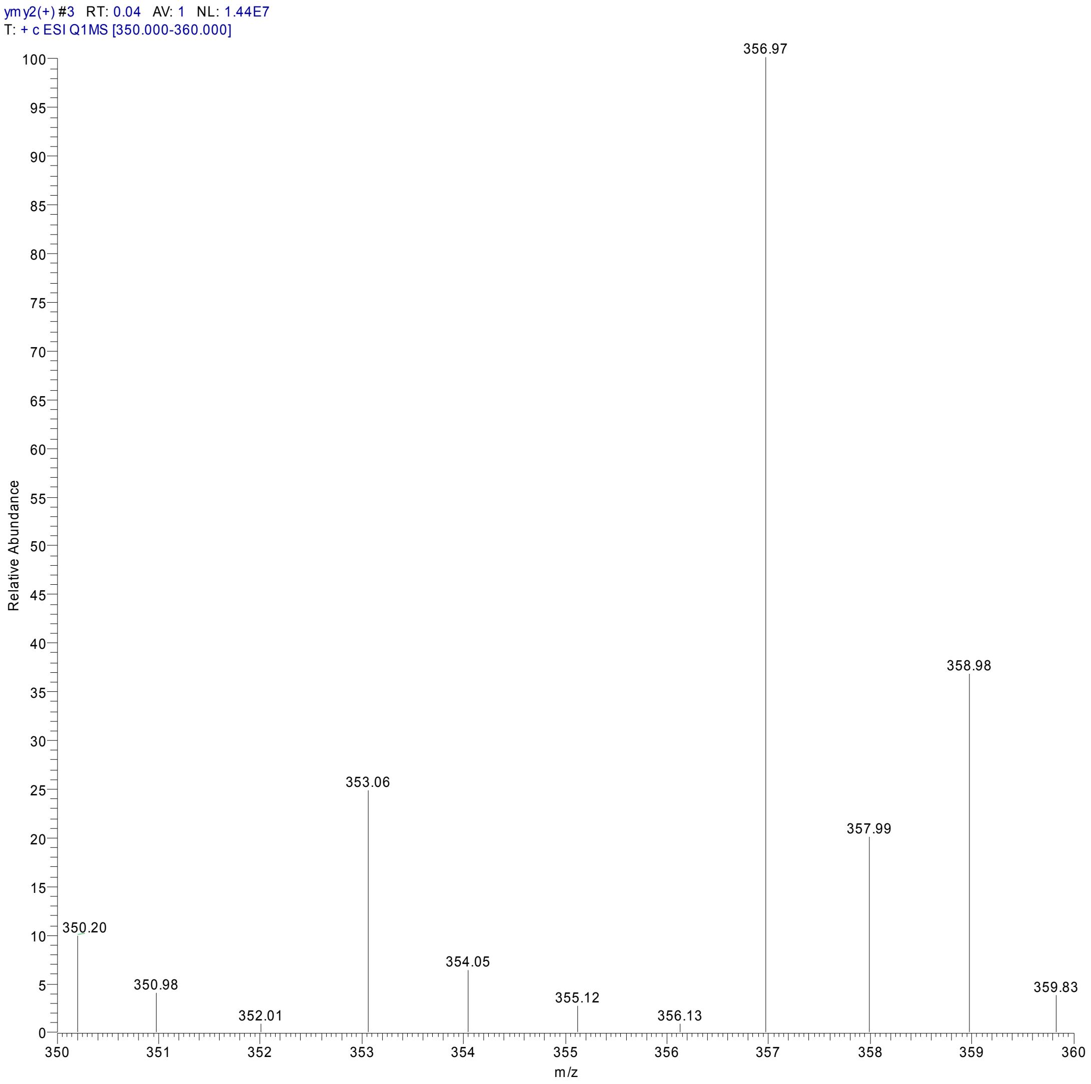

Figure S19. MS spectrum of compound **5b** (+).

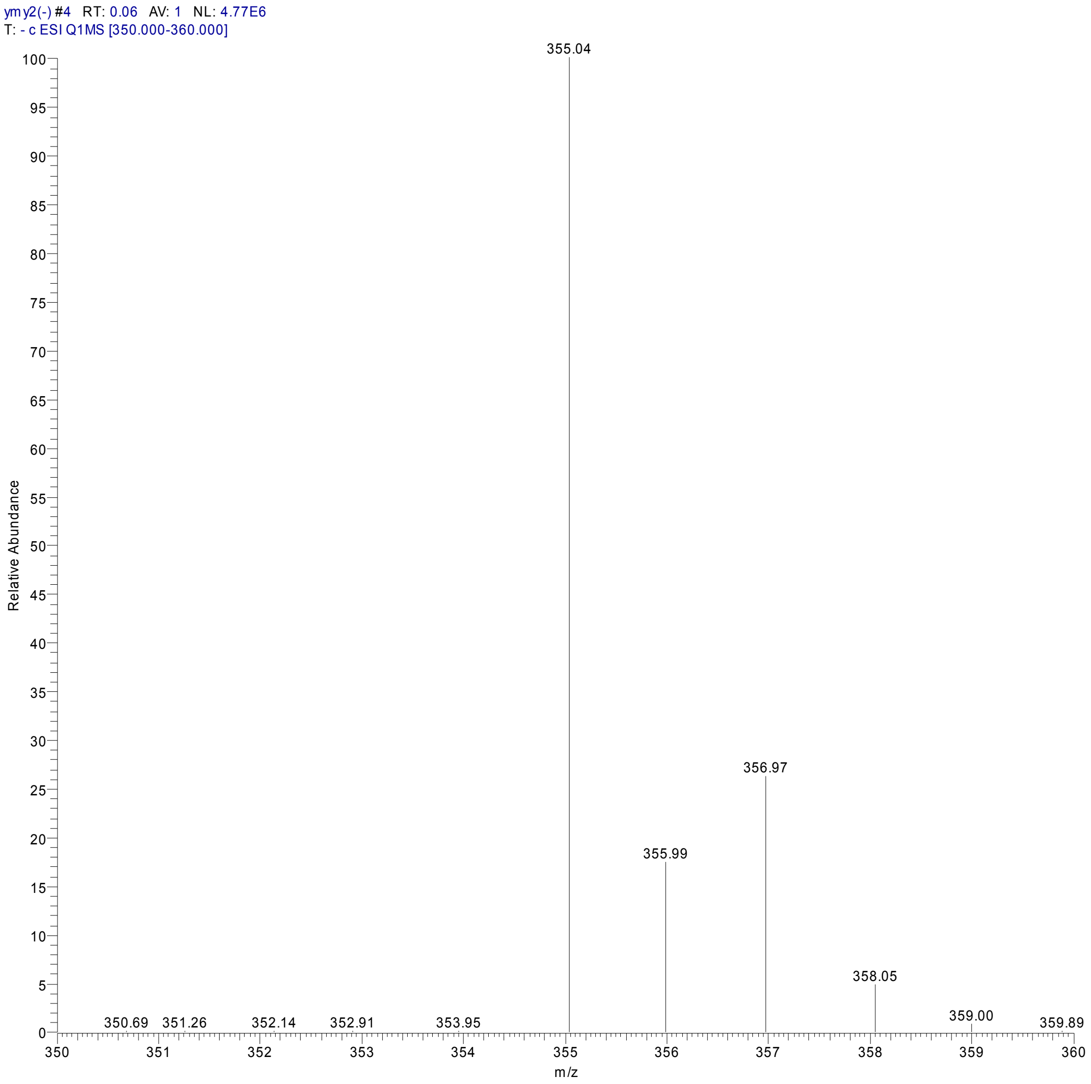

Figure S20. MS spectrum of compound **5b** (−).

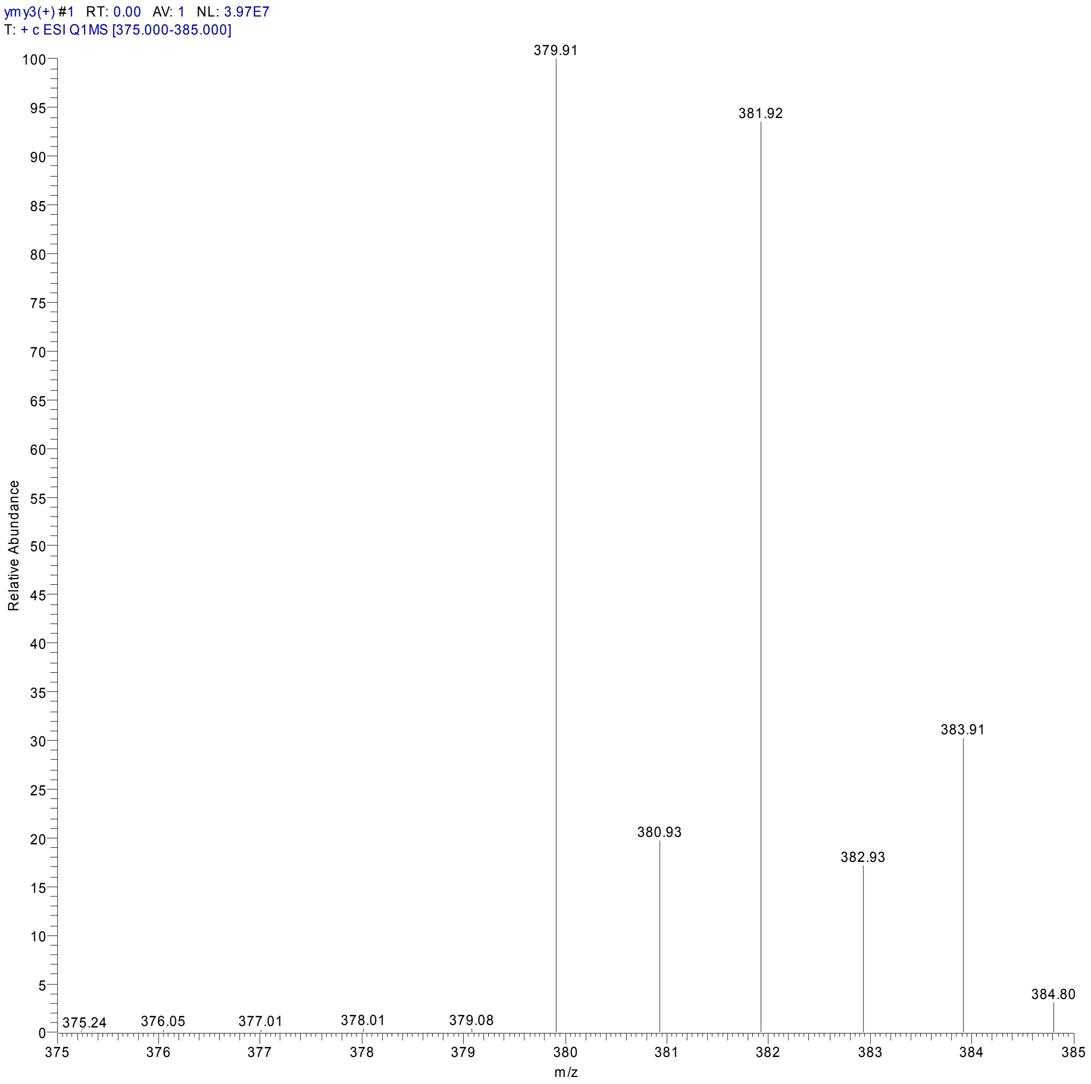

Figure S21. MS spectrum of compound **5c** (+).

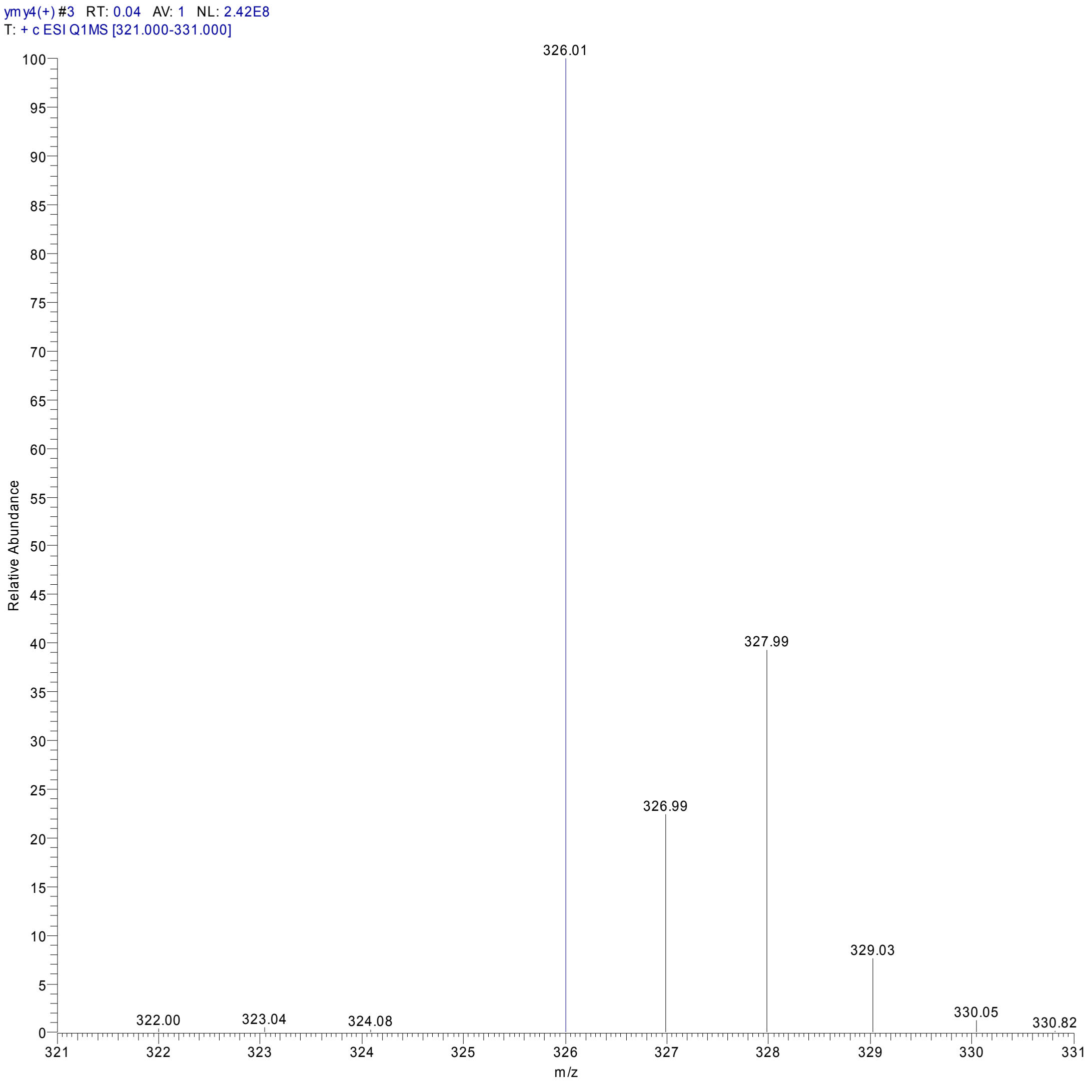

Figure S22. MS spectrum of compound **5d** (+).

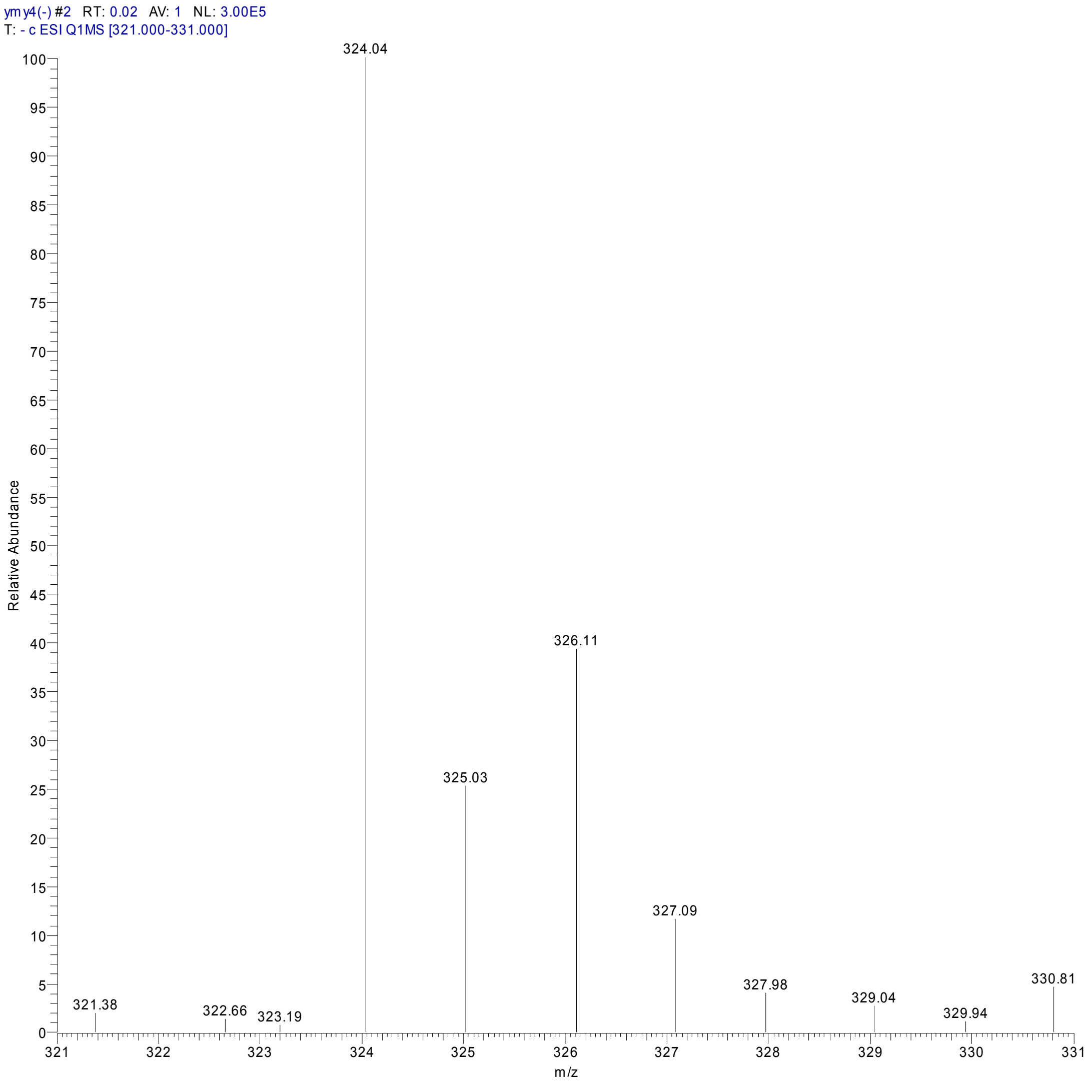

Figure S23. MS spectrum of compound **5d** (−).

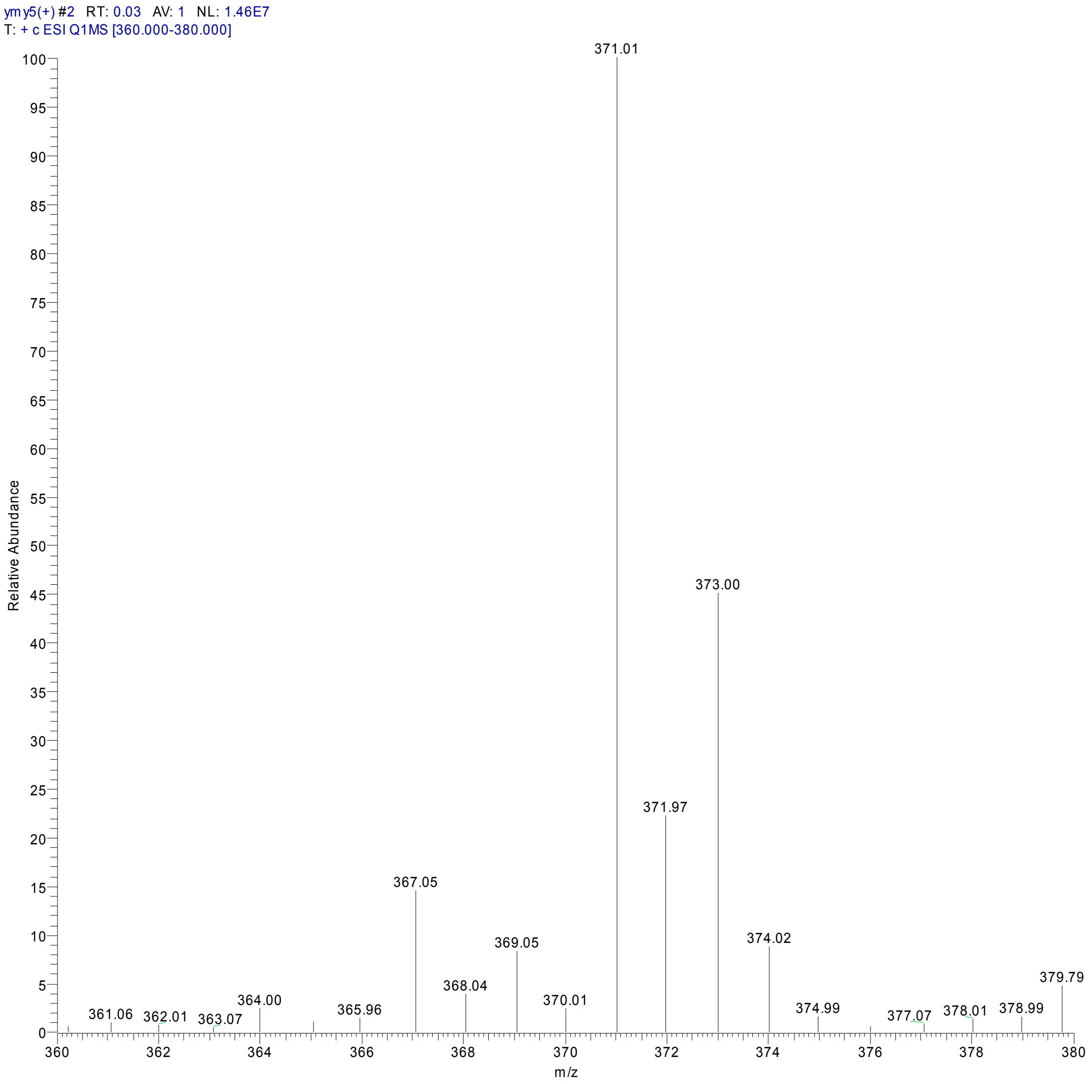

Figure S24. MS spectrum of compound **5e** (+).

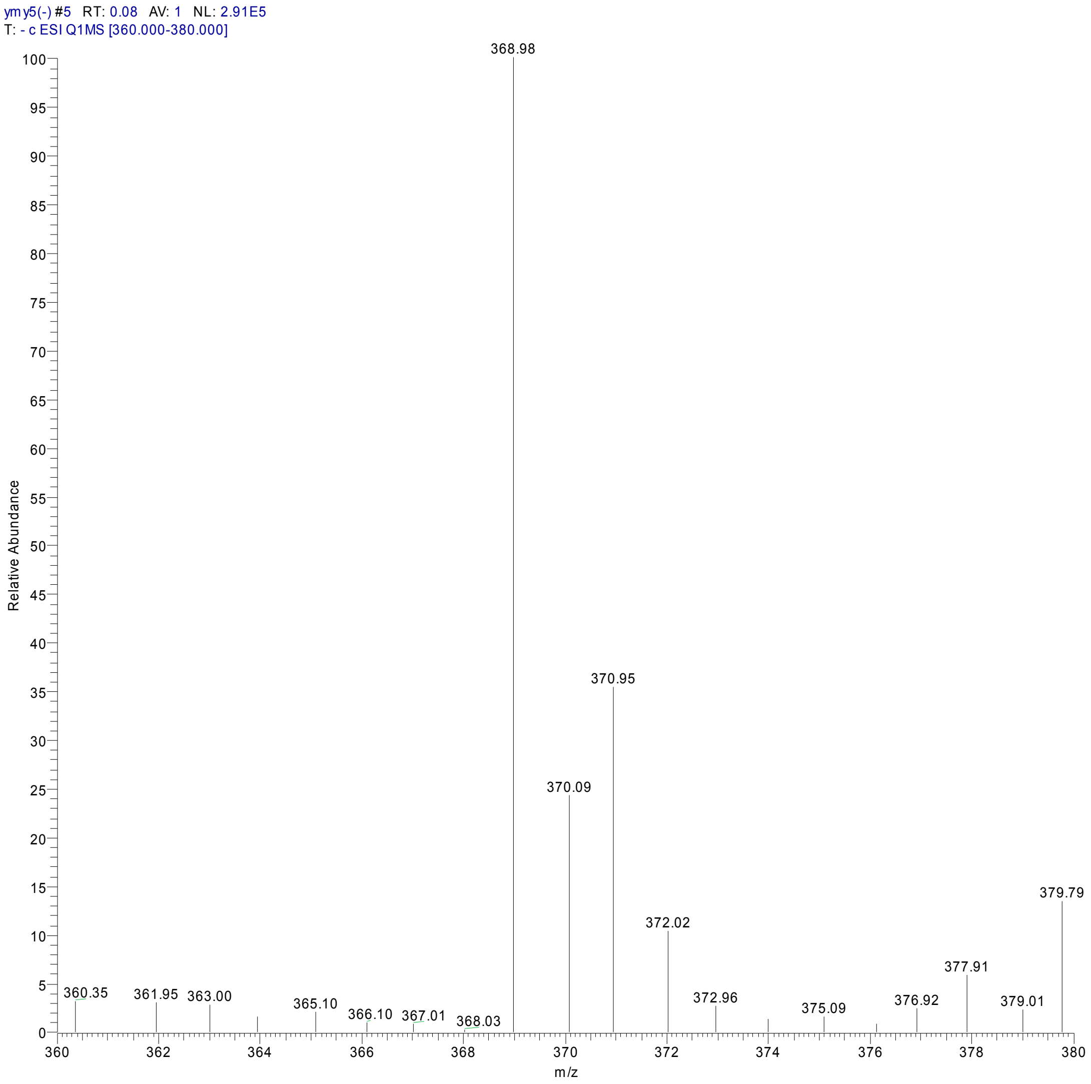

Figure S25. MS spectrum of compound **5e** (−).

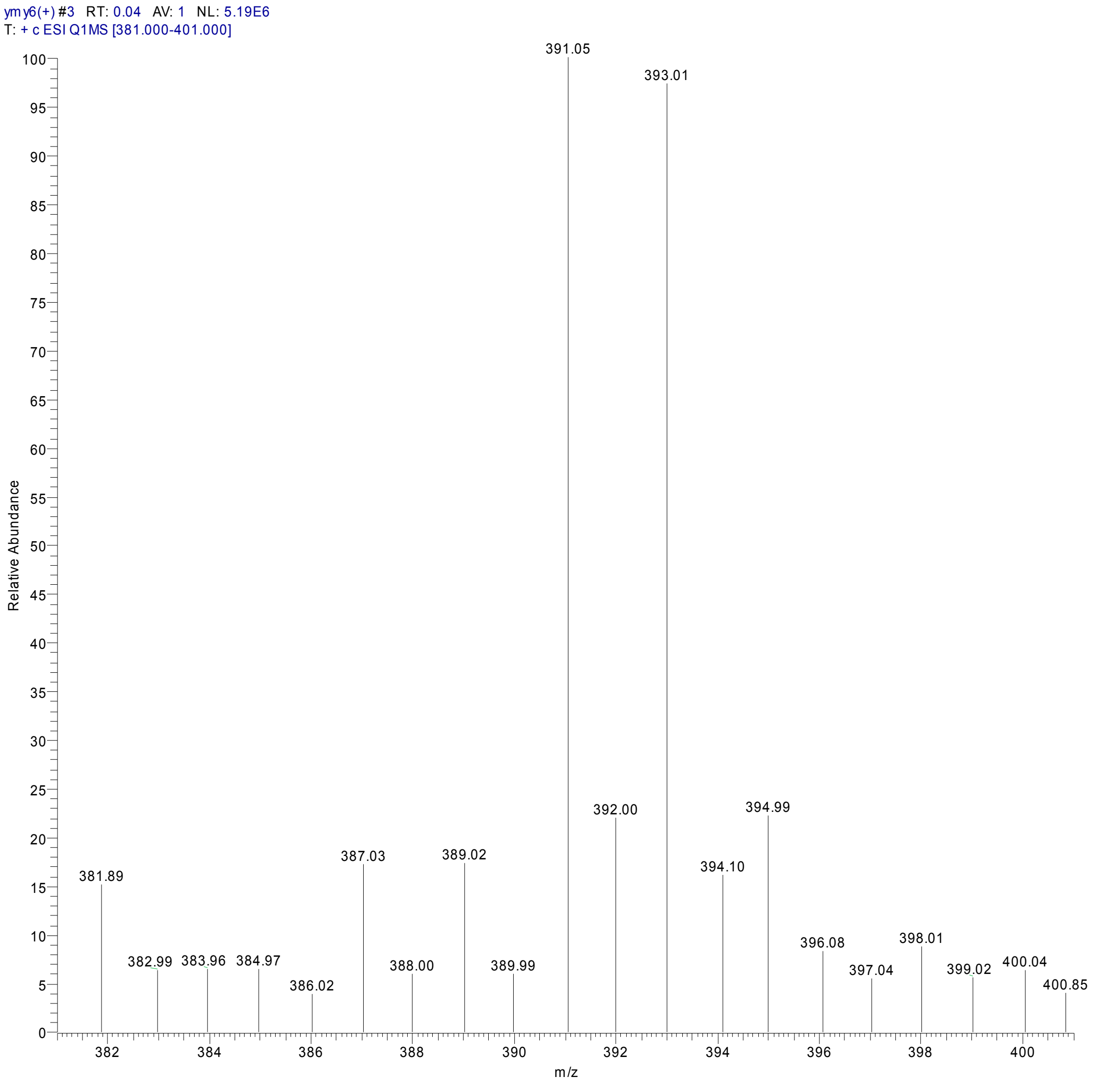

Figure S26. MS spectrum of compound **5f** (+).

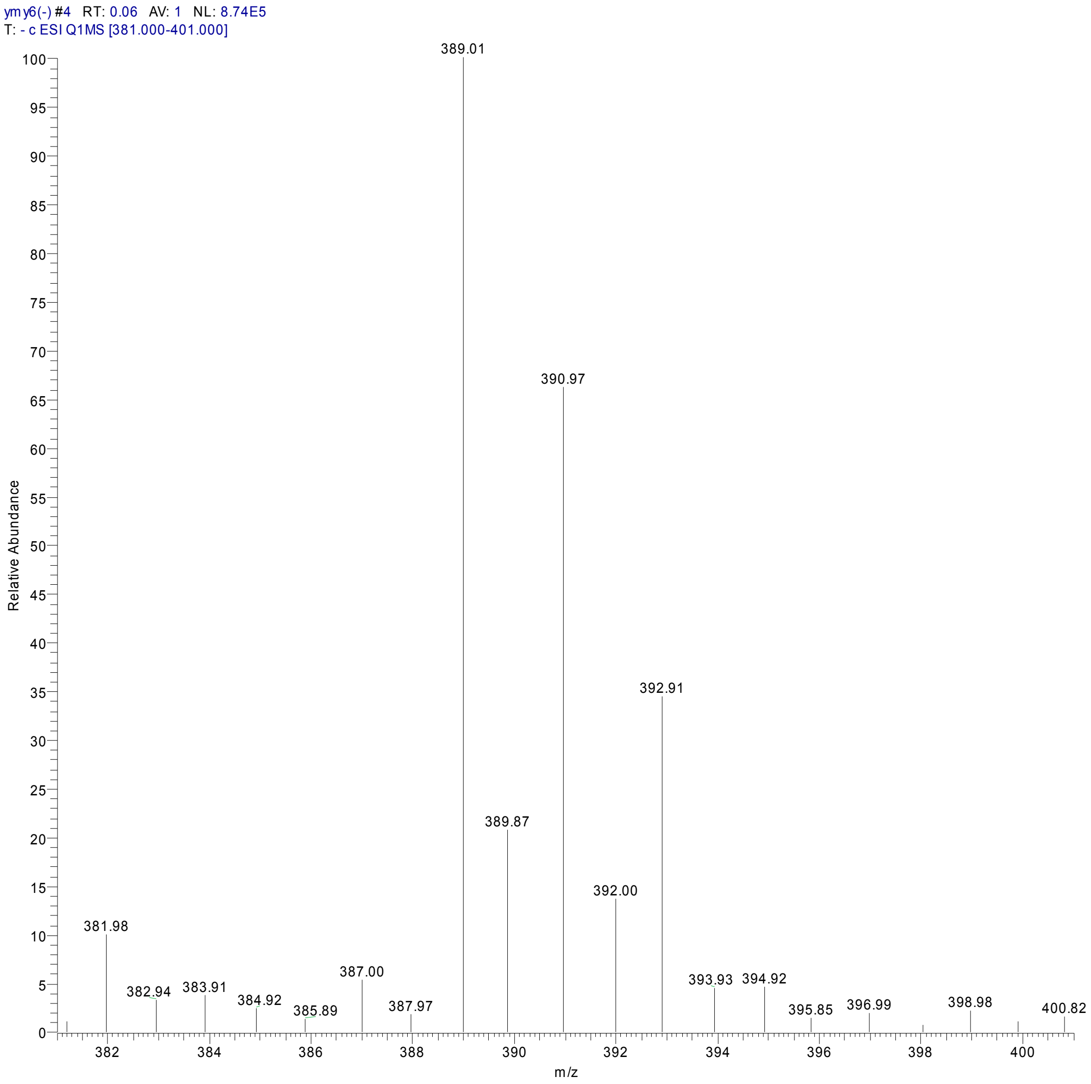

Figure S27. MS spectrum of compound **5f** (−).

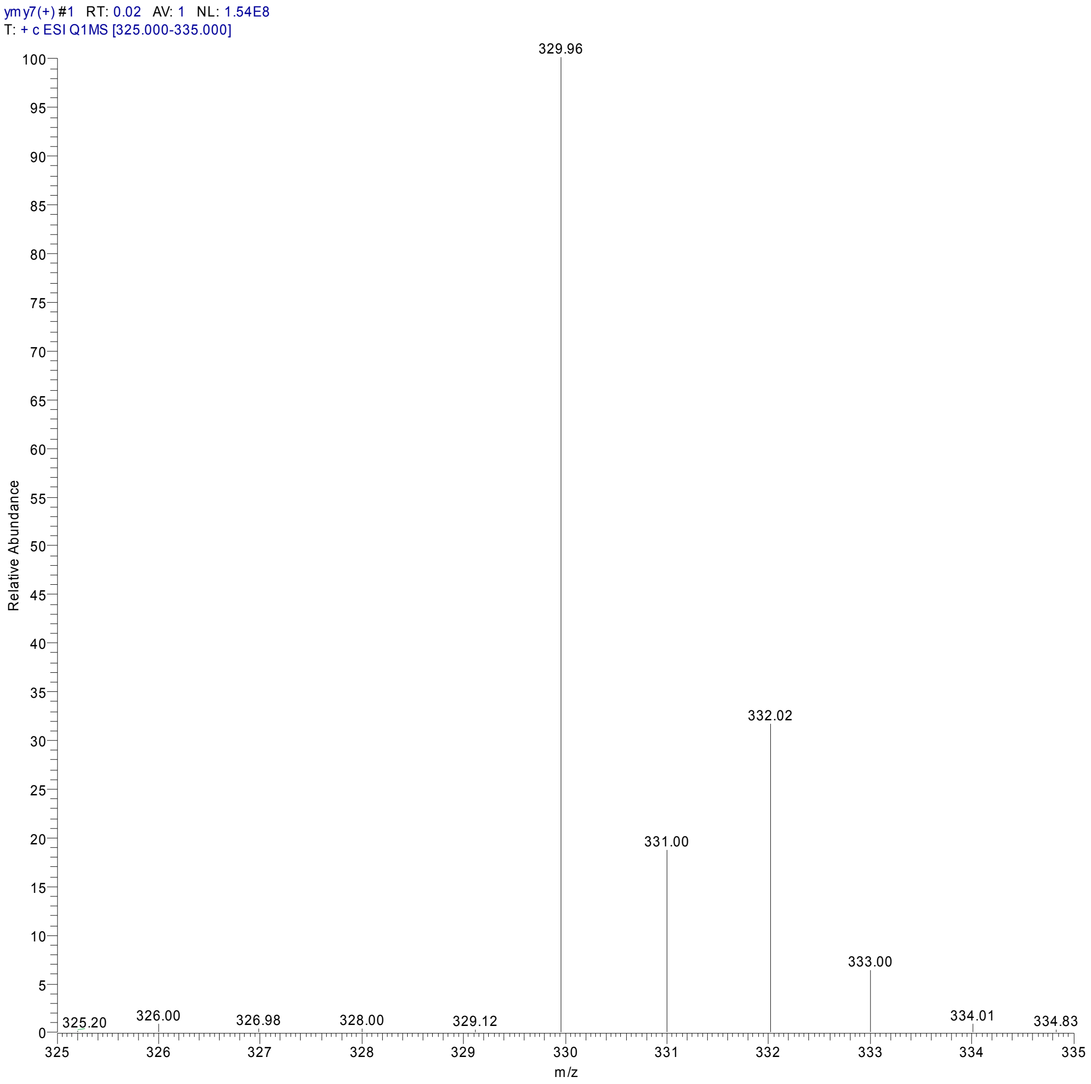

Figure S28. MS spectrum of compound **5g** (+).

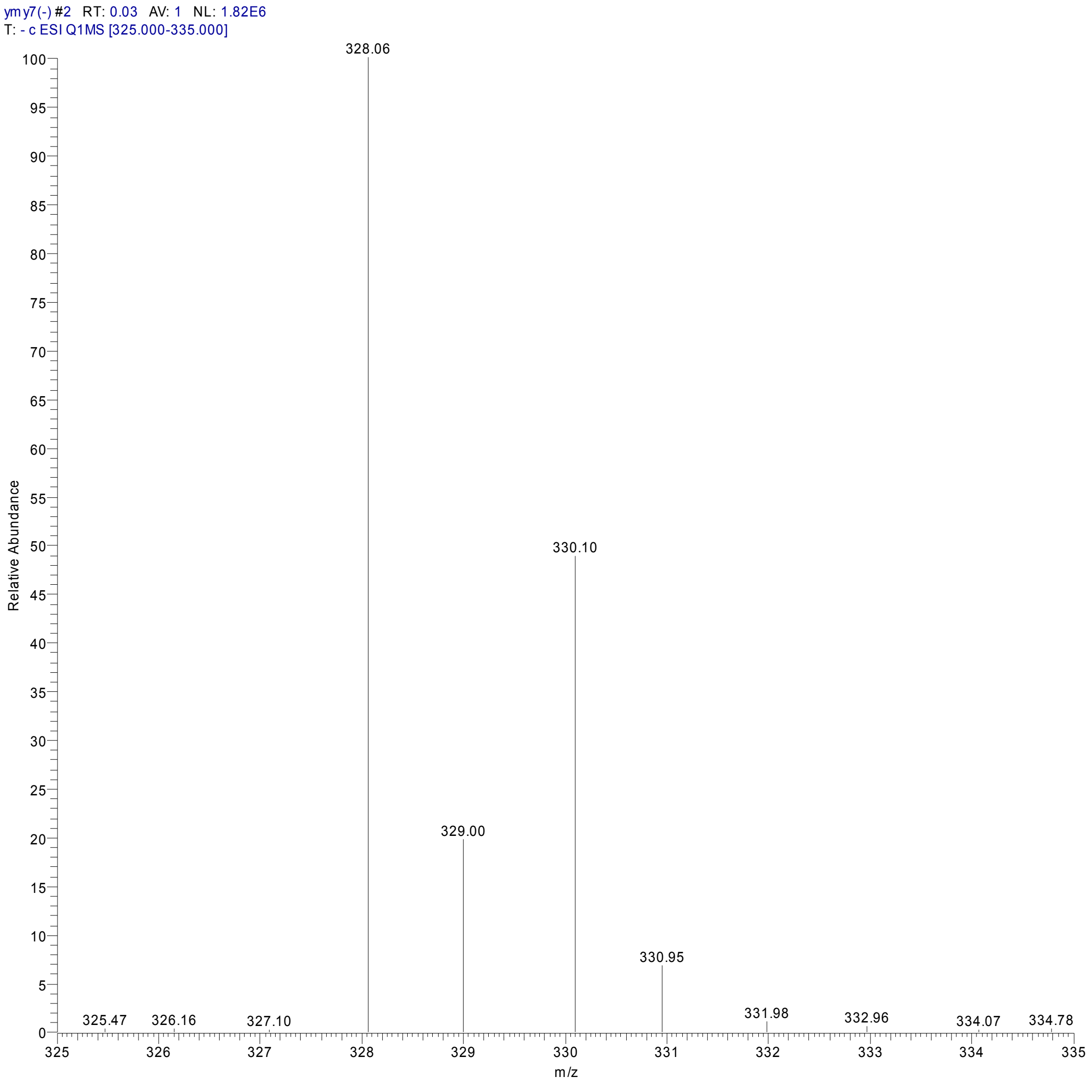

Figure S29. MS spectrum of compound **5g** (−).

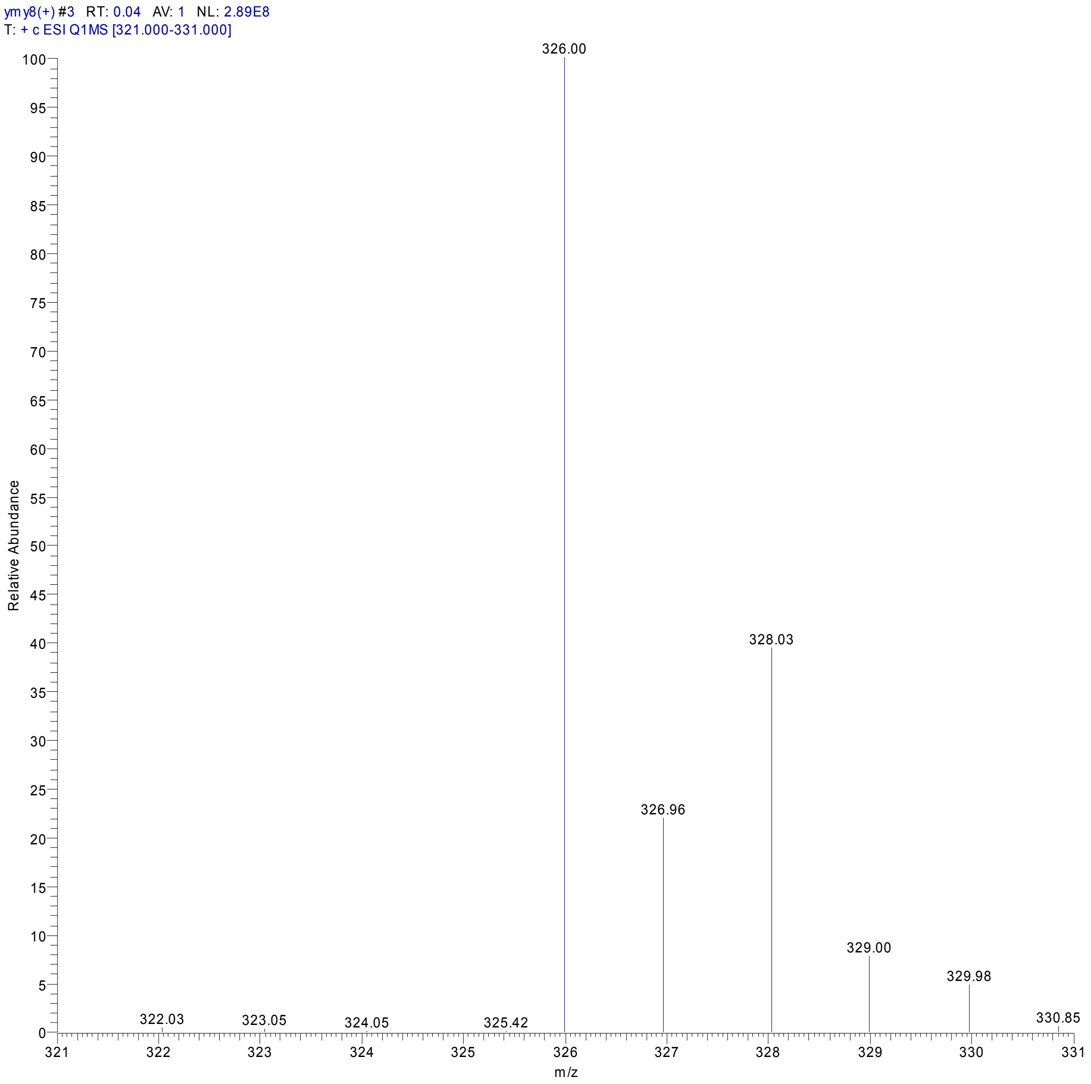

Figure S30. MS spectrum of compound **5h** (+).

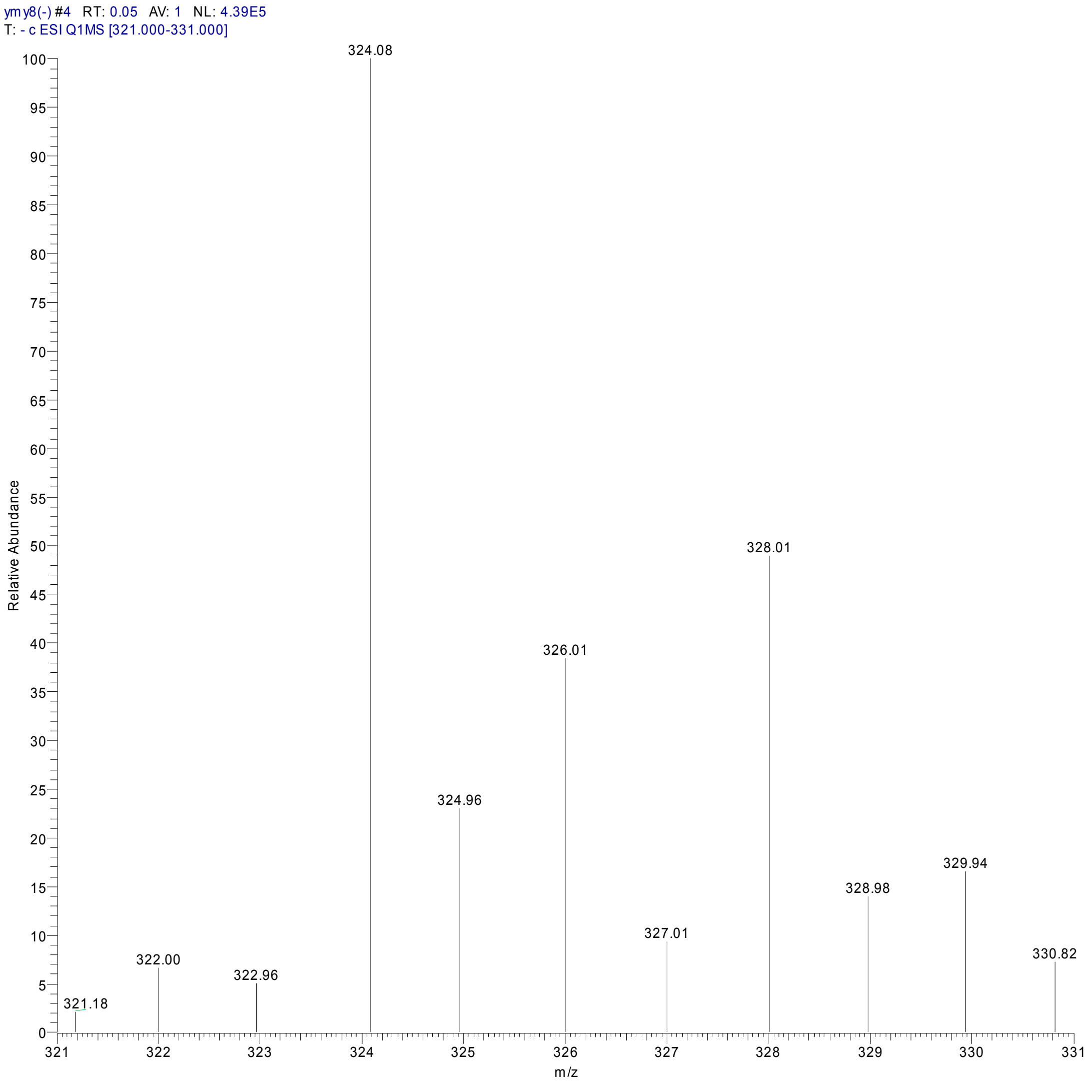

Figure S31. MS spectrum of compound **5h** (−).

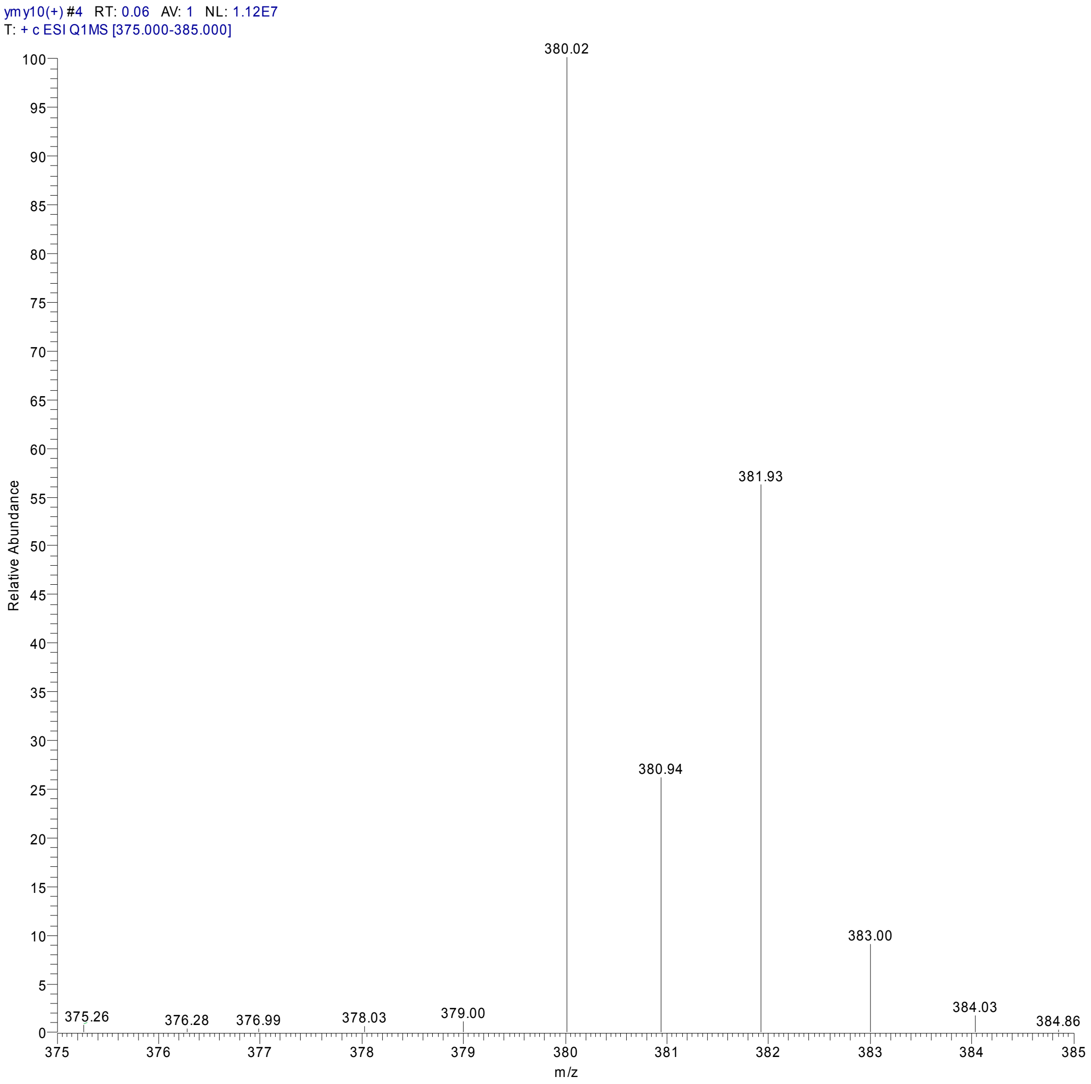

Figure S32. MS spectrum of compound **5i** (+).

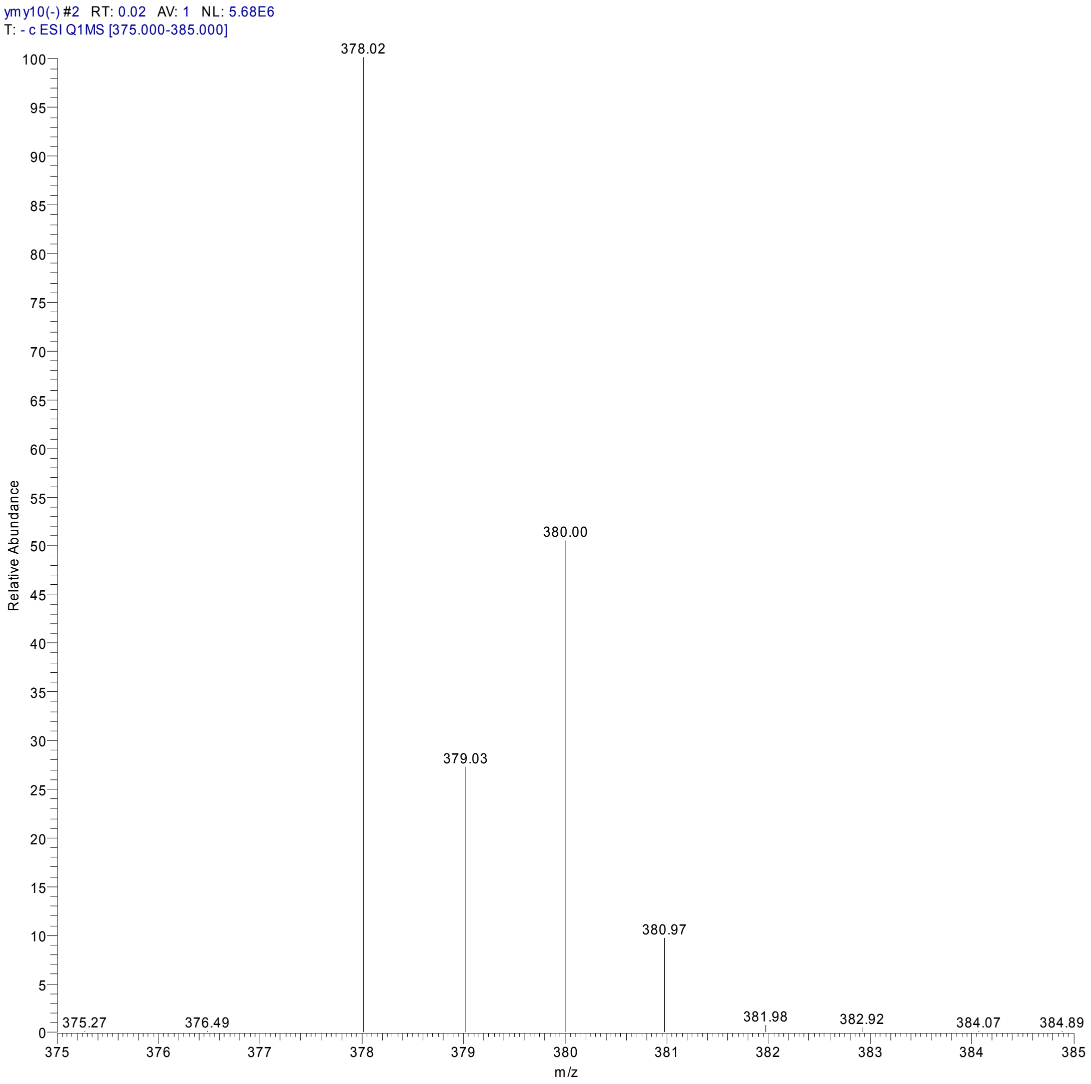

Figure S33. MS spectrum of compound **5i** (−).

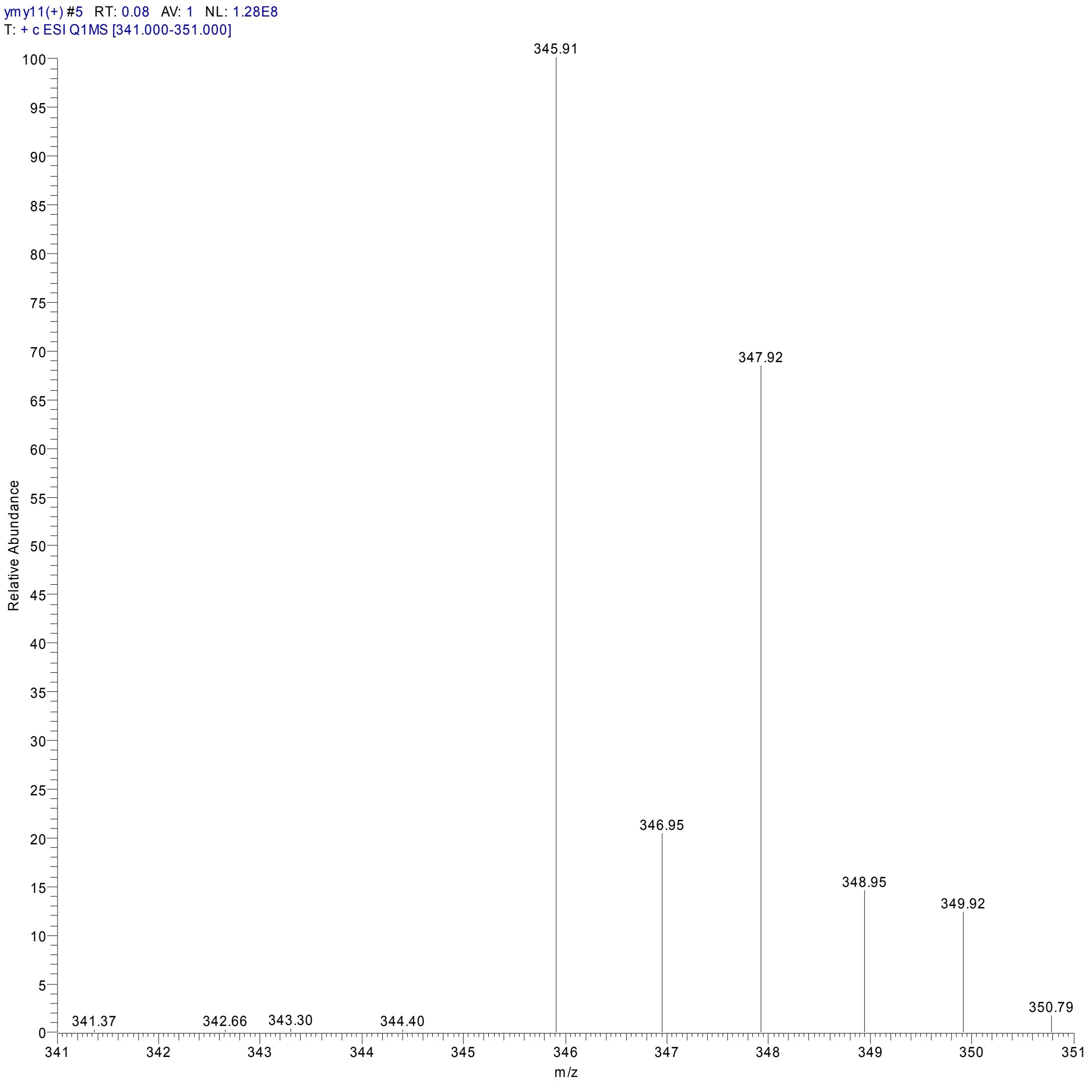

Figure S34. MS spectrum of compound **5j** (+).

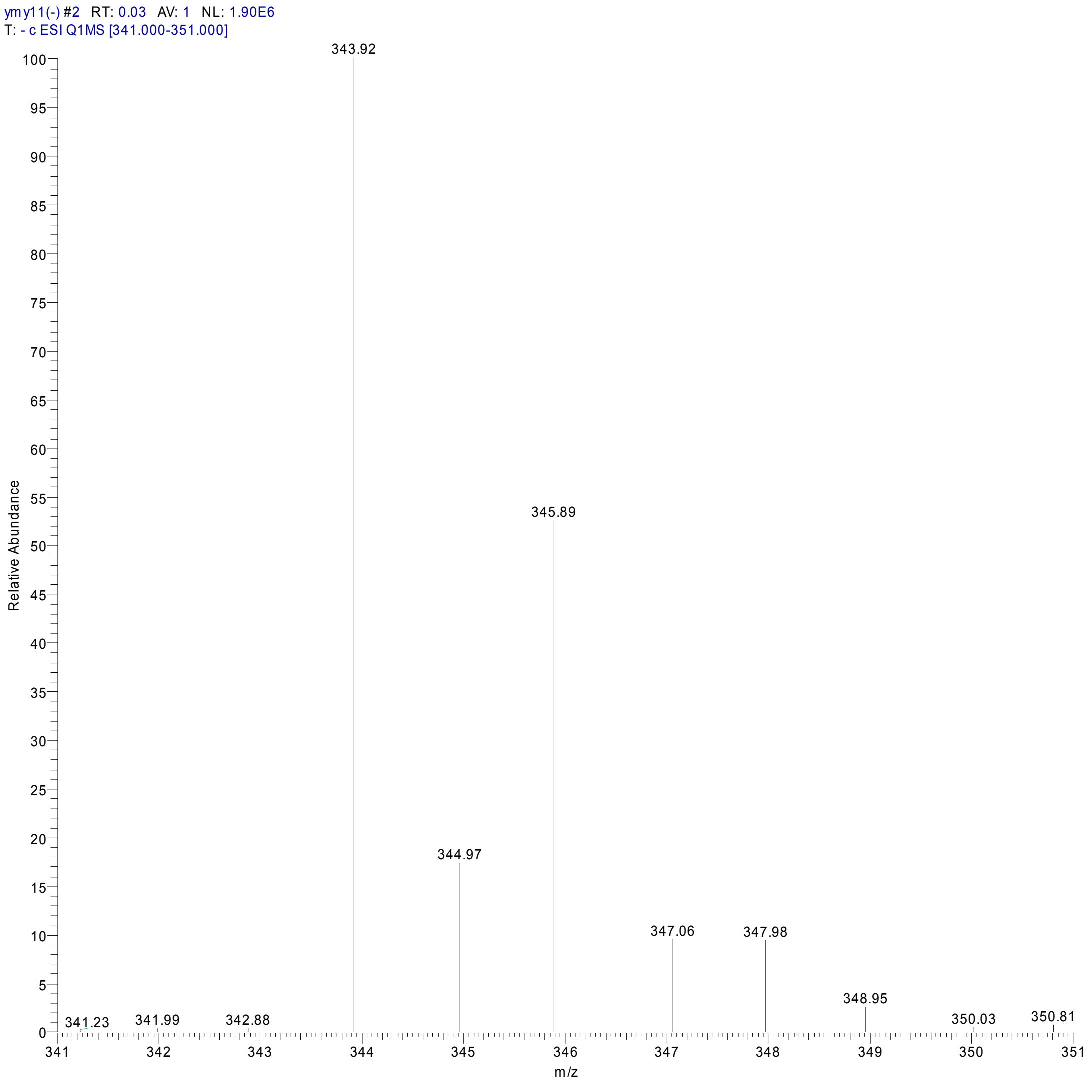

Figure S35. MS spectrum of compound **5j** (−).

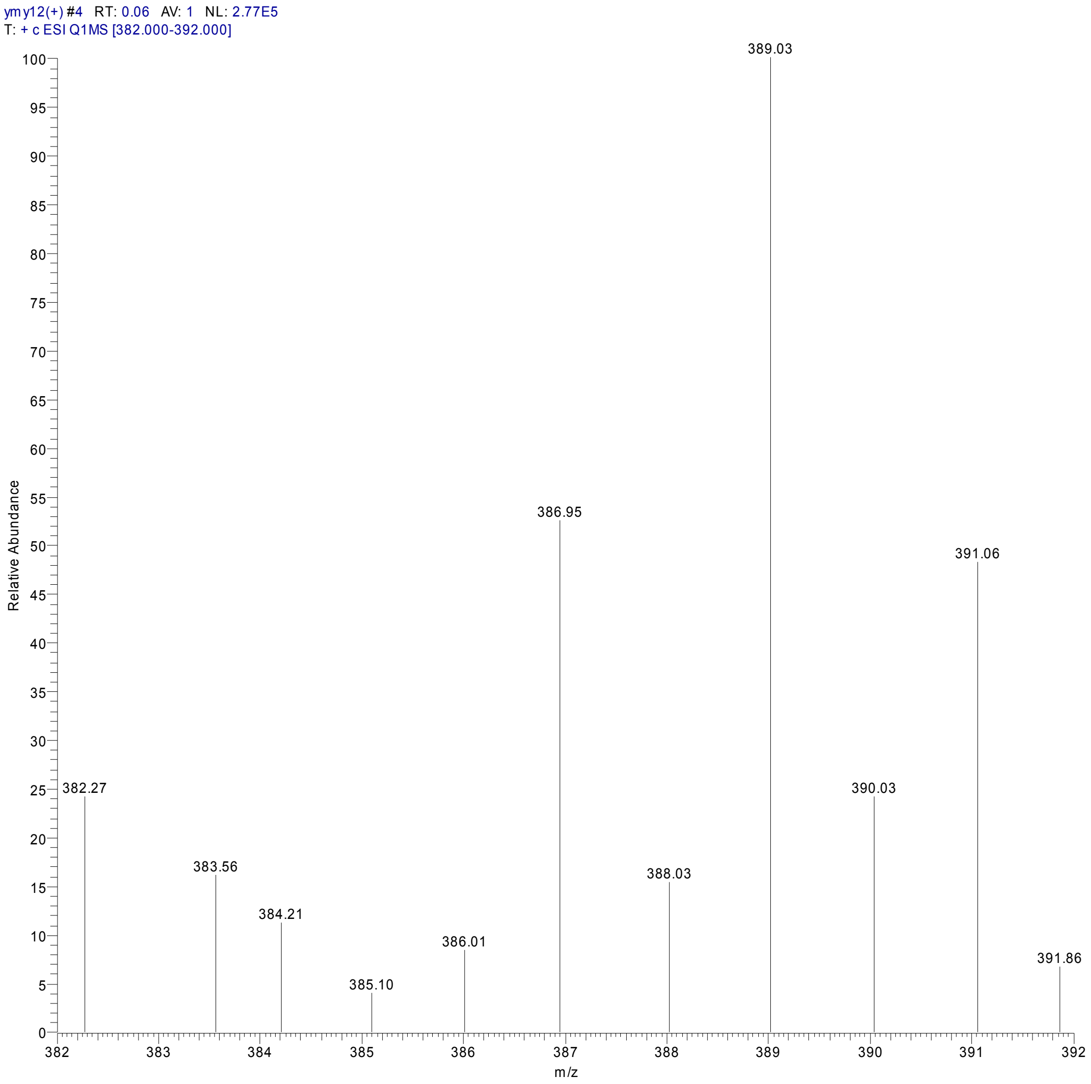

Figure S36. MS spectrum of compound **5k** (+).

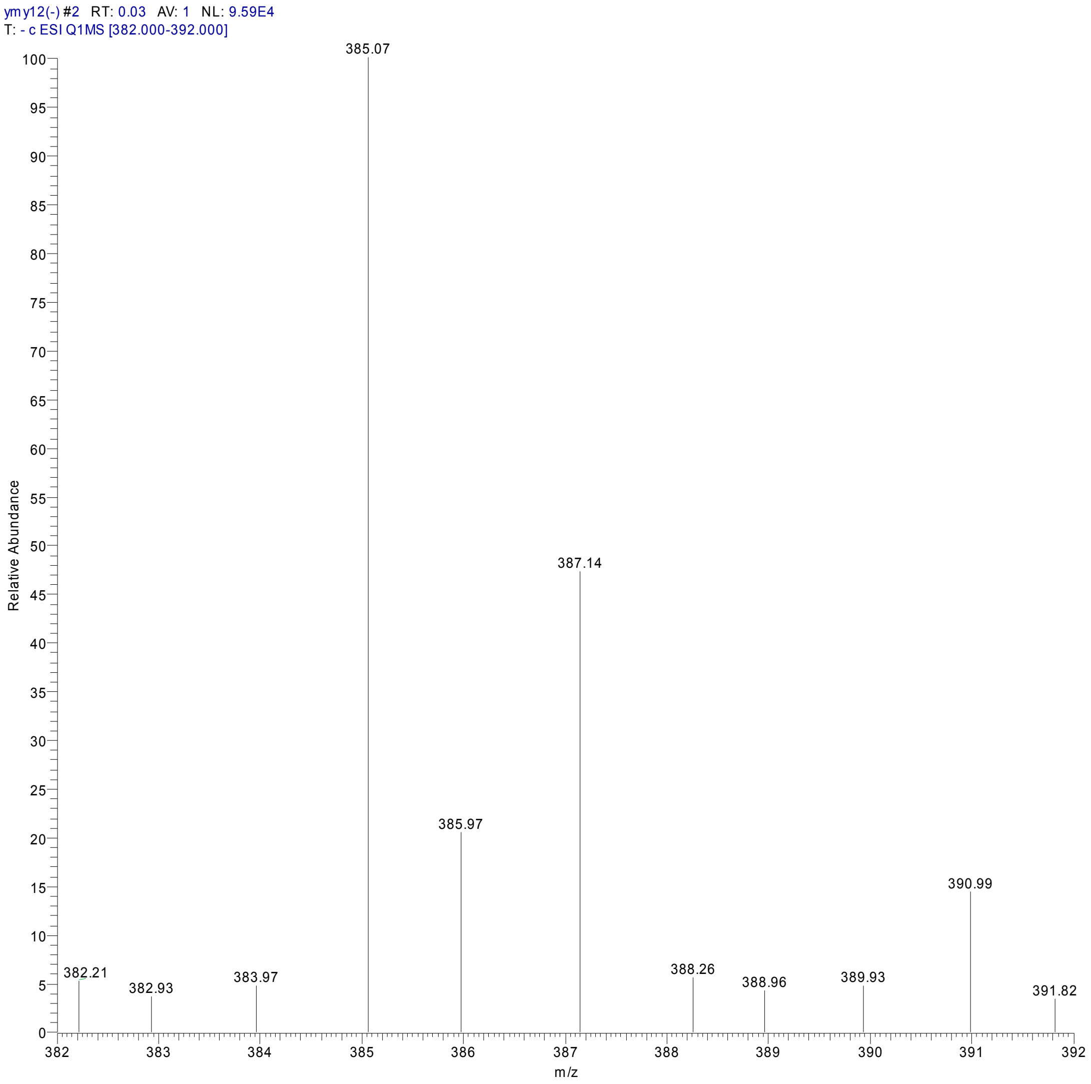

Figure S37. MS spectrum of compound **5k** (−).

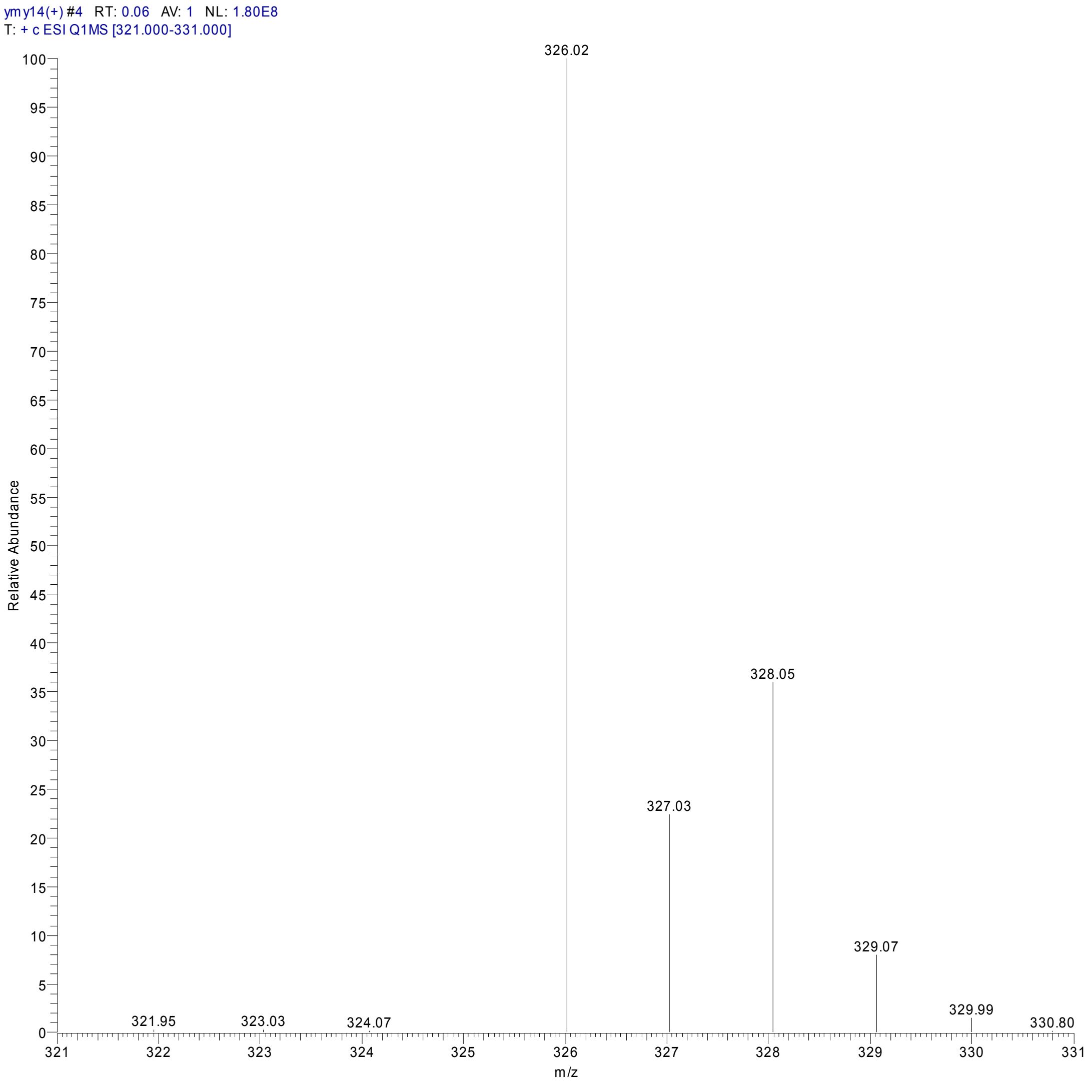

Figure S38. MS spectrum of compound **5l** (+).

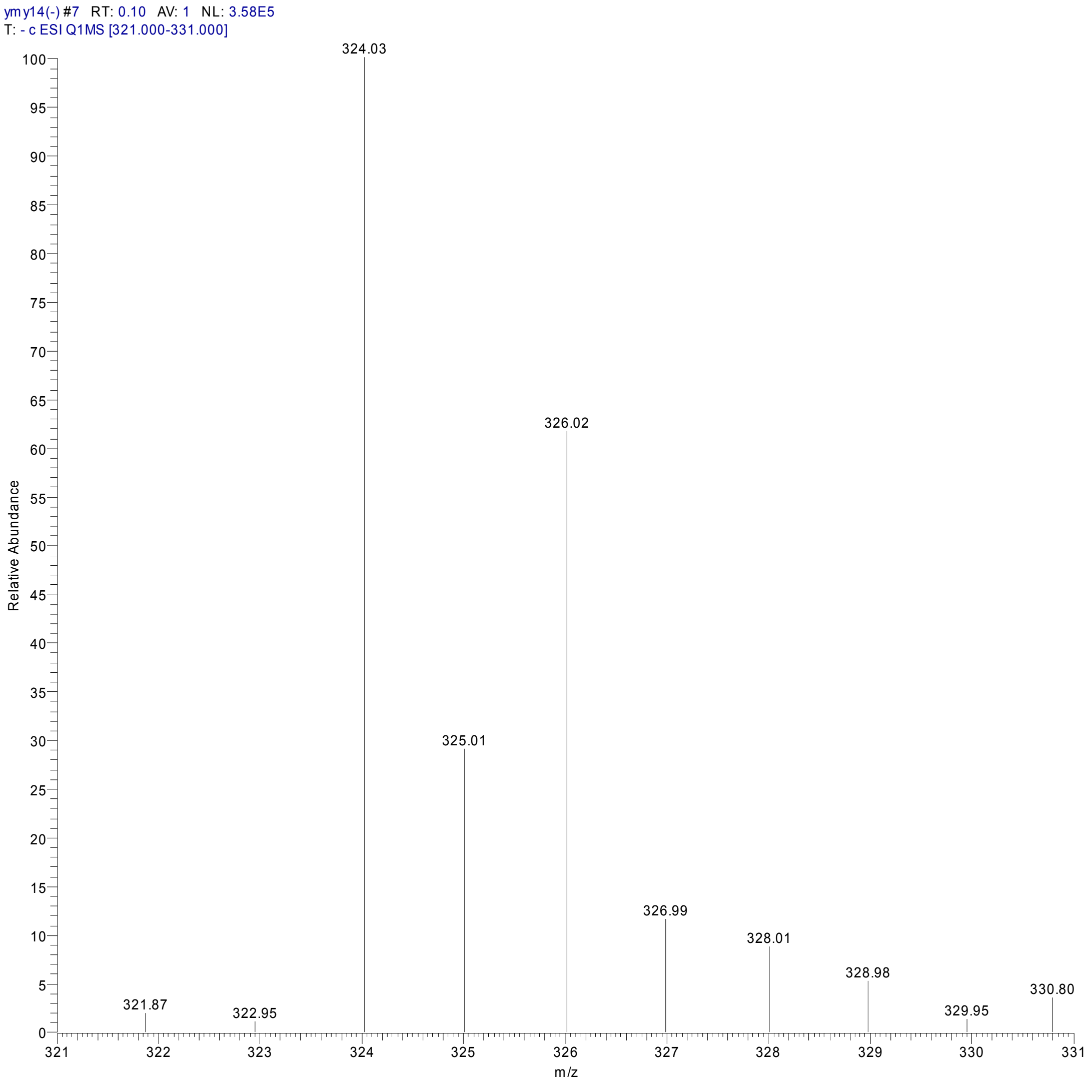

Figure S39. MS spectrum of compound **5l** (−).

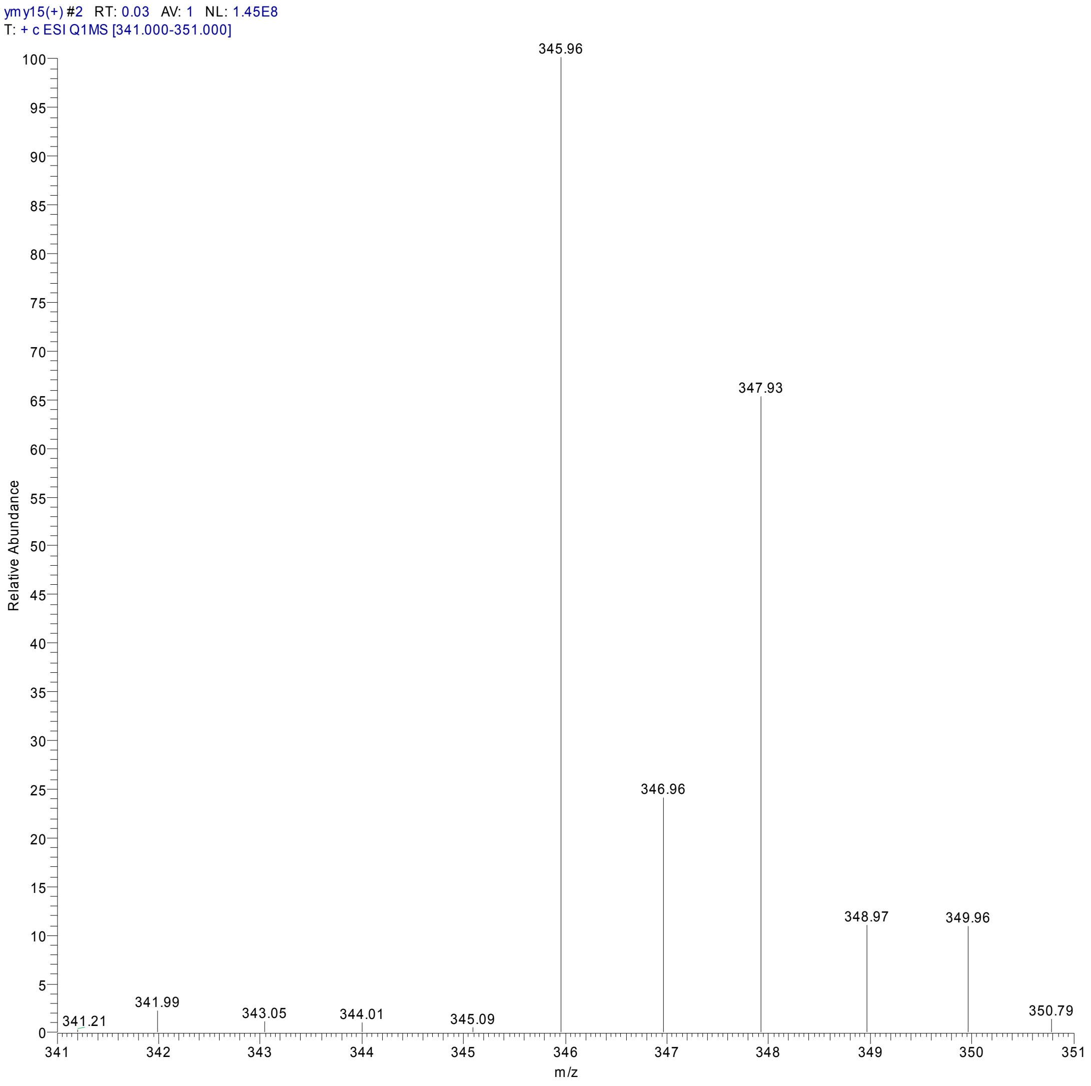

Figure S40. MS spectrum of compound **5m** (+).

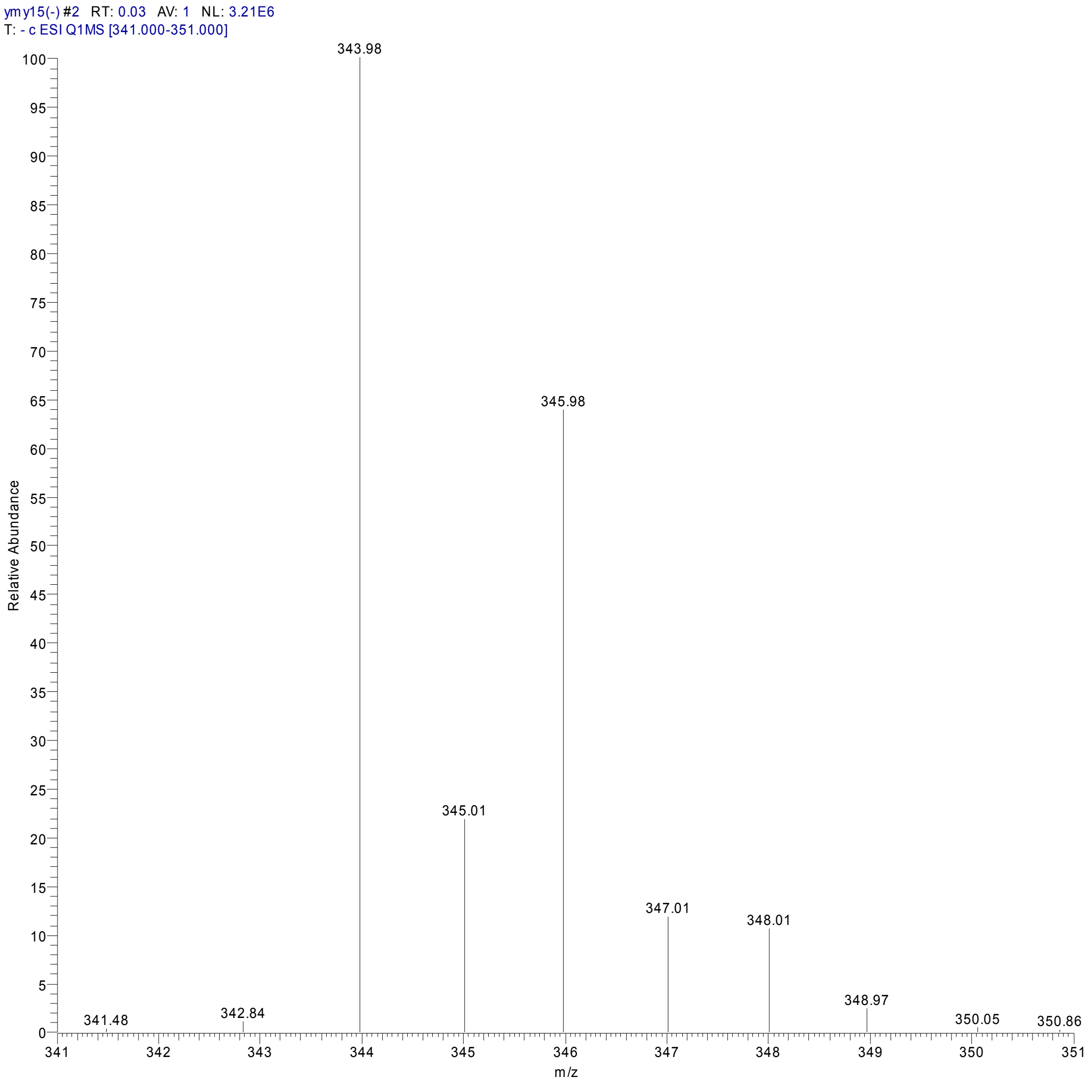

Figure S41. MS spectrum of compound **5m** (−).
